# Allele frequencies at recessive disease genes are mainly determined by pleiotropic effects in heterozygotes

**DOI:** 10.1093/genetics/iyag150

**Published:** 2026-06-15

**Authors:** Jonathan Judd, Jeffrey P Spence, Nikhil Milind, Linda Kachuri, John S Witte, Jonathan K Pritchard

**Affiliations:** Department of Genetics, Stanford University, Stanford, CA 94305, United States; Department of Genetics, Stanford University, Stanford, CA 94305, United States; Institute for Human Genetics, University of California SanFrancisco, San Francisco, CA 94158, United States; Department of Epidemiology & Biostatistics, University of California SanFrancisco, San Francisco, CA 94158, United States; Department of Genetics, Stanford University, Stanford, CA 94305, United States; Department of Epidemiology and Population Health, Stanford University, Stanford, CA 94305, United States; Stanford Cancer Institute, Stanford University School of Medicine, Stanford, CA 94305, United States; Department of Genetics, Stanford University, Stanford, CA 94305, United States; Department of Epidemiology and Population Health, Stanford University, Stanford, CA 94305, United States; Stanford Cancer Institute, Stanford University School of Medicine, Stanford, CA 94305, United States; Department of Genetics, Stanford University, Stanford, CA 94305, United States; Department of Biology, Stanford University, Stanford, CA 94305, United States

**Keywords:** population genetics, recessive diseases, quantitative genetics

## Abstract

The classic theory of mutation–selection balance predicts the equilibrium frequency of genetic variation under negative selection. The model predicts a simple relationship between the total frequency of deleterious variants, mutation rate, and strength of selection, with different functions for recessive and (co)dominant genes. In this study, we investigate whether genes associated with human recessive disorders fit the predictions of this classic model. By comparing observed frequencies of loss-of-function (LoF) variants to those expected under mutation–selection balance we find that, for nearly all recessive genes, the observed frequencies are too low to be explained by purely recessive selection. Analyzing the effects of heterozygous LoFs on quantitative traits from the UK Biobank, we find that recessive disease genes have widespread quantitative effects in heterozygotes. Together, these results suggest that for a large proportion of recessive disease genes, the majority of selection acting on pathogenic mutations appears to operate through heterozygotes. We conclude that very few human genes follow the classic model of recessive mutation–selection balance.

## Teaser

Classical genetics predicts that genes causing recessive diseases should harbor pathogenic variants at relatively high frequencies, since natural selection only removes them when two copies are inherited. Judd et al. tested this across 796 human recessive disease genes using population-scale sequencing data. For nearly all of them, observed variant frequencies were far too low to fit the purely recessive model, even accounting for inbreeding and genetic drift. UK Biobank data revealed that these genes have widespread quantitative trait effects in single-copy carriers. These findings suggest natural selection on recessive disease genes operates primarily through heterozygotes, fundamentally challenging a long-standing textbook model.

## Introduction

A century ago, J.B.S. Haldane developed the now-classic theory of “mutation–selection balance,” which predicts the equilibrium frequency of deleterious variants (Haldane 1927, 1937). The key insight of mutation–selection balance is that, at equilibrium, the rate at which new deleterious variants are generated in a gene by mutations must exactly offset the rate at which variants are removed by natural selection.

In the last decade, advances in whole-exome and whole-genome sequencing have enabled sequencing of hundreds of thousands of healthy people (Lek et al. 2016; Karczewski et al. 2020; Chen et al. 2024; Guo et al. 2024). Consistent with the predictions of mutation–selection balance, these studies show that important genes are highly depleted of functional variants such as loss-of-function (LoF) mutations (Lek et al. 2016; Karczewski et al. 2020; Guo et al. 2024). This observation has motivated a great deal of work on estimating the strength of selection against LoF heterozygotes (here denoted $s_{\mathrm{het}}$) for each gene, usually assuming additive models of selection (Cassa et al. 2017; Fuller et al. 2019; Agarwal et al. 2023; Zeng et al. 2024).

However, the standard additive model does not apply to fully recessive genes. In this case, selection acts only in homozygotes; since homozygotes are rare compared to heterozygotes, the classic mutation–selection balance theory predicts that pathogenic variants should be found at much higher frequencies in recessive genes than in dominant or codominant genes.

It is generally believed that the recessive version of mutation–selection balance should apply to a wide variety of autosomal recessive diseases. Recessive diseases, including cystic fibrosis, sickle cell anemia, and Tay–Sachs disease (Lonergan et al. 2001; Fernandes Filho and Shapiro 2004; Ong and Ramsey 2023; McKusick 2024), occur when patients inherit pathogenic mutations that abrogate function in both copies of a particular gene.

While significant progress has been made on estimating $s_{\mathrm{het}}$, estimating selection coefficients in homozygotes (denoted $s_{\mathrm{hom}}$) remains challenging due to the difficulty of disentangling heterozygous effects from homozygous effects in population genetic studies, and due to the rarity of homozygotes and survivorship bias in observational studies (Amorim et al. 2017; Cassa et al. 2017; Balick et al. 2022).

Here, we take a different approach, simply asking whether the frequencies of putative pathogenic mutations in annotated recessive genes are compatible with recessive mutation–selection balance. After addressing several technical challenges we conclude that, for the vast majority of annotated recessive genes, the frequencies of pathogenic mutations are too low to be explained by a fully recessive model.

To explain this, we propose that most recessive disease genes also have meaningful phenotypic effects in heterozygotes—and that these pleiotropic effects are the main target of selection. At a mechanistic level, we can expect that heterozygous mutations would often reduce gene expression or functionality by 50%; this in turn might affect molecular phenotypes, such as chromatin accessibility, with cascading quantitative effects on organism-level traits (Boyle et al. 2017; Zschocke et al. 2023; Milind et al. 2024; Domingo et al. 2025). Consistent with this prediction, we find that LoF mutations in genes annotated as being fully recessive frequently have significant effects on quantitative traits measured by UK Biobank (UKB) (Barton et al. 2022; Heyne et al. 2023; Fridman et al. 2025). These heterozygous effects are likely too small to be recognized as clinical conditions, and yet strong enough to be subject to negative selection.

In summary, we report here that the frequencies of pathogenic mutations in nearly all annotated recessive genes are too low to be explained by Haldane’s classical model of recessive mutation–selection balance. Instead, we propose that most selection at these genes is due to selection acting against quantitative effects in heterozygotes.

## Methods

### Identifying recessive genes

An initial list of 1,183 recessive genes was curated by previous studies (Blekhman et al. 2008; Berg et al. 2013; Amorim et al. 2017) by inclusion in Online Mendelian Inheritance in Man (OMIM), a comprehensive compendium of human genes and genetic phenotypes (Amberger et al. 2015), and manual curation through other resources such PubMed and Gene Reviews (Adam et al. 1993; White 2020). To increase the likelihood that the list of recessive genes only affected traits through autosomal recessive inheritance, the initial list of recessive genes was cross-referenced against the OMIM database in December 2024, and any genes with nonautosomal recessive inheritance were excluded in order to produce the most updated list of 796 genes associated with recessive traits.

### Gene data

Gene-level information about LoF variants, including allele frequencies and mutation locations, were retrieved from the gnomAD database (Karczewski et al. 2020). For this study, we used gnomAD v4.0 genomic information from 705,148 unrelated individuals in the Non-Finnish European (NFE), Finnish (FIN), African (AFR), and South Asian (SAS) ancestry groups. We defined LoFs as those predicted to cause stop-gain, splice-acceptor, and splice-donor effects using the Variant Effect Predictor (VEP; v85) (McLaren et al. 2016). When calculating gene-level LoF allele frequencies ($p_{\mathrm{LoF}}$), we summed allele frequencies for LoFs within the gene, excluding variants with an allele frequency >0.005 to exclude LoFs with potential bioinformatic errors (Zeng et al. 2024; Stolyarova et al. 2025). (Our overall conclusions are robust to excluding this filter.) For each gene, $p_{\mathrm{LoF}}$ was calculated separately for each population. This variant inclusion/exclusion criteria potentially excludes many pathogenic missense and LoF variants that may lead to a recessive disease in these genes (Molotkov et al. 2024). However, the chosen variants are known to be highly likely to stop gene function and increases our confidence in examining pathogenic variation and its effect in recessive mutation–selection balance (Cummings et al. 2020; Karczewski et al. 2020; Stolyarova et al. 2025). Total mutation rates for each gene were calculated by considering all possible LoF-causing point mutations and using a mutation rate that accounts for trinucleotide context and methylation status at CpGs as described in Cummings et al. (2020) and Karczewski et al. (2020). Final population-specific $p_{\mathrm{LoF}}$s and total mutation rates along with the previously determined recessive gene status were used to visualize additive and recessive mutation–selection balance.

### Adjusting mutation–selection balance for missing variation

We wanted to estimate the proportion of pathogenic variants that might be excluded from the types of variants we considered above: stop-gain, splice-donor-disrupting, and splice-acceptor-disrupting point mutations. In particular, we identified the percentage of pathogenic variation in 25 well-known recessive disease genes represented by these classes of variants (Jackson et al. 2018; Xiao and Lauschke 2021). Using ClinVar (Landrum et al. 2025) to identify pathogenic and likely pathogenic short variation in recessive disease genes, we analyzed genes including *HBB*, *CFTR*, *PAH*, *ATP7B*, *MUTYH*, *HEXA*, *GBA1*, *USH2A*, and *SMN1*, which are the genes associated with sickle cell anemia, cystic fibrosis, phenylketonuria (PKU), Wilson disease, MUTYH-associated gastric cancer, Tay–sachs disease, Gaucher disease, Usher syndrome, and spinal muscular atrophy, respectively (Lonergan et al. 2001; Fernandes Filho and Shapiro 2004; Castellani 2013; Nagral 2014; Garbade et al. 2019; Kumar et al. 2020; Castiglione and Möller 2022; Mercuri et al. 2022; Hemker et al. 2025). After using the ClinVar-provided molecular consequences to identify LoFs and non-LoFs, we calculated the percentage of unique pathogenic variation that were predicted to cause stop gain, disrupt a splice donor, or disrupt a splice acceptor. For each gene, the inverse of these percentages was calculated as a conversion factor to adjust for potential missing non-LoF pathogenic variation, and we adjusted the visualizations of recessive mutation–selection balance by increasing $p_{\mathrm{LoF}}$ by the average of the calculated conversion factors and the square root total mutation rates by the square root of the average of the conversion factors.

To determine the conversion factors required so that 50 and 90 of recessive disease genes fit the recessive mutation–selection balance model, we identified the recessive disease gene whose ratio between the LoF allele frequency, $p_{\mathrm{LoF}}$, and square root total mutation rate, $\sqrt{\mu L}$, is at the 50th and 10th percentile of all recessive disease gene ratios, respectively. From the ratios of the genes at the 50th and 10th percentile, the conversion factors are the square of the inverse of the ratio for the gene (Equation (1)).


(1) $$\text{Conversion-Factor}={\left(\frac{1}{\text{ratio}}\right)}^{2}={\left(\frac{1}{\frac{\;p_{\mathrm{LoF}}}{\sqrt{\mu L}}}\right)}^{2}={\left(\frac{\sqrt{\mu L}}{p_{\mathrm{LoF}}}\right)}^{2}$$

### Mutation–selection balance with inbreeding

Along with the classic mutation–selection balance model (Equation (8)), we extended a model by Nei that calculates the expected equilibrium $p_{\mathrm{LoF}}$ in a finite population (Supplementary Appendix A) (Nei 1968) to account for the effect of inbreeding depression on recessive genes in a more realistic population (Equation (2)):


(2) $$E[p_{\mathrm{path}}]=\frac{\sum_{k=0}^{\infty }\frac{{\left(-4Ns_{\mathrm{hom}}\varphi /(1+\varphi \right)}^{k}\Gamma \left(2N\mu /(1+\varphi )+(k+1)/2\right)}{k!{\left(2Ns_{\mathrm{hom}}(1-\varphi )/(1+\varphi )\right)}^{2N\mu /(1+\varphi )+(k+1)/2}}}{\sum_{\ell =0}^{\infty }\frac{{\left(-4Ns_{\mathrm{hom}}\varphi /(1+\varphi \right)}^{\ell }\Gamma \left(2N\mu /(1+\varphi )+\ell /2\right)}{\ell !{\left(2Ns_{\mathrm{hom}}(1-\varphi )/(1+\varphi )\right)}^{2N\mu /(1+\varphi )+\ell /2}}}$$

With these two models, we can predict $p_{\mathrm{LoF}}$ under recessive lethal selection for different total mutation rates and inbreeding values, providing a lower bound for values of $p_{\mathrm{LoF}}$ consistent with a purely recessive model.

### Simulating populations with varied inbreeding coefficients

In order to assess the fit of Equations (1) and (3) for varying levels of inbreeding assuming recessive lethality, we used SLiM v4.3 to generate simulated population data (Haller and Messer 2023). All simulated populations contained 20,000 individuals and possessed a single recessive lethal gene that accumulated LoF variants over 200,000 generations. We generated 125 simulated populations each with 25 varying and evenly spaced total mutation rates ([0–0.005]) and inbreeding coefficients ([0, 1/64, 1/32, 1/16, 1/8]). After simulating and calculating $p_{\mathrm{LoF}}$ for each population, we generated theoretical model values for the same parameters using Equations (8) and (2).

The simulated data were plotted over the predicted lower bounds for LoF allele frequency under different levels of inbreeding (Equations (8) and (2)) to assess fit. Additionally, empirical recessive gene data was compared to both models under varying inbreeding conditions to determine the required level of inbreeding for recessive genes to be consistent with purely recessive mutation–selection balance.

### Burden tests

We used REGENIE (Mbatchou et al. 2021) to perform gene-level burden tests on 161 quantitative traits available in UKB (Bycroft et al. 2018). For the whole-genome regression, which is the first step in REGENIE, SNPs from the genotyping array were pruned with a 1,000 variant sliding window with 100 variant shifts and an $R^{2}$ threshold of 0.9. SNPs were then filtered to have MAF <1 (to reduce the impact of misannotation and LD with other functional SNPs), genotype missingness <10, and Hardy–Weinberg equilibrium test *p*-value $>10^{-15}$. For LoF burden tests, we used the following covariates: age, sex, age by sex, age squared, 15 genotyping PCs, 20 rare variant PCs, and WES batch. After whole-genome regression, we used the second step of REGENIE to perform burden tests using the whole-exome sequencing data. We used the LOFTEE plugin in Ensembl’s VEP to annotate high-confidence LoF sites (McLaren et al. 2016; Karczewski et al. 2020). We further filtered any annotated LoF site with MAF <1 using a variable–frequency filter across genes based on their constraint, as described previously (Milind et al. 2024; Zeng et al. 2024). For each gene, we filtered to individuals that carried at most one LoF variant, ensuring that burden tests were performed using only LoF heterozygotes. Phenotypes were rank-inverse-normal transformed in both the first and second step. The same covariates were used in both steps.

To generate a list of genetically uncorrelated traits, we used previously released estimates of genetic correlation based on common variants from the UKB (http://www.nealelab.is/uk-biobank/). Any traits with genetic correlation greater than 0.75 were clustered together, and traits with the most signal based on bias-corrected average squared burden effect size were picked from the clusters to generate a list of 21 uncorrelated traits.

### Measuring burden effects across gene constraint

The estimated heterozygous gene constraint was calculated for all genes to determine whether a positive relationship exists between heterozygous selection and heterozygous trait effects. Prior to constraint estimation, genes falling below the 25th percentile of total mutation rate were excluded across both recessive and nonrecessive genes to reduce noise introduced by genes with limited mutational targets. For nonrecessive genes, heterozygous gene constraint was estimated using GeneBayes-predicted $s_{\mathrm{het}}$ values, which provide well-calibrated estimates regardless of the observed $p_{\mathrm{LoF}}$.

For recessive genes, $s_{\mathrm{het}}$ was estimated by dividing the gene’s total mutation rate by $p_{\mathrm{LoF}}$ as modeled by additive mutation–selection balance (Equation (5)). Recessive genes with $p_{\mathrm{LoF}}=0$ following the mutation rate filter were retained rather than excluded, as neutrality expectations predict at least approximately 10 segregating LoF variants for such genes; observed $p_{\mathrm{LoF}}$ values near zero therefore likely reflect strong purifying selection rather than an absence of informative data, rendering removal of these genes inappropriate.

Next, for each gene, we estimated the mean squared burden effect size $E[{\hat{\gamma }}^{2}]$ from the Regenie output. Specifically, because the expectation of the squared estimated effect size satisfies


(3) $$E[{\hat{\gamma }}^{2}]\approx \gamma^{2}+{\text{SE}}^{2},$$

an unbiased estimate of the true squared effect size was obtained by subtracting the squared standard error of the test from the squared estimated effect size:


(4) $${\hat{\gamma^{2}}}_{\mathrm{unbiased}}={\hat{\gamma }}^{2}-{\text{SE}}^{2}.$$

Bias-corrected squared effect sizes were then averaged by gene to provide each gene an estimated constraint value and mean squared burden test effect size. All genes were subsequently segregated into deciles based on estimated gene constraint, and average squared burden test effect sizes within each bin were further stratified by recessive gene status. Final decile effect sizes were separated by recessive classification and plotted to visualize the relationship between heterozygous constraint and heterozygous effect size.

### Sensitivity analysis of the burden enrichment–constraint relationship

To assess the robustness of the observed relationship between heterozygous gene constraint and burden effect size to potential misestimation of $s_{\mathrm{het}}$ in recessive genes, we performed a sensitivity analysis in which recessive gene $s_{\mathrm{het}}$ values were scaled by multiplicative factors of ×1, ×0.75, ×0.5, and ×0.25 prior to constraint binning. For each scaling scenario, precalculated $s_{\mathrm{het}}$ estimates for recessive genes were multiplied by the corresponding factor, and recessive and nonrecessive genes were pooled together to assign constraint decile bins via joint ranking of the scaled recessive and unscaled nonrecessive $s_{\mathrm{het}}$ values. The nonrecessive reference curve was held fixed by computing its decile assignments from the original unscaled joint distribution (×1). To reduce noise, the analysis was restricted to genes in the top 75% of all genes by total mutation rate. Loess curves of mean bias-corrected squared burden effect size across constraint deciles were estimated separately for the nonrecessive reference and for recessive genes under each scaling factor.

### Estimating heterozygous selection fraction

To estimate a lower bound on heterozygous gene constraint for recessive genes, we simplified the mutation–selection balance model ($2\mu L=2p_{\mathrm{path}}(1-p_{\mathrm{path}})s_{\mathrm{het}}+2p_{\mathrm{path}}^{2}s_{\mathrm{hom}}$) to compute $s_{\mathrm{het}}$ given the gene’s $p_{\mathrm{path}}$ and total mutation rate, assuming recessive lethality ($s_{\mathrm{hom}}=1$). Details on the estimator are provided in Supplementary Appendix A. This $s_{\mathrm{het}}$ estimate serves as the constraint measure for recessive genes in the burden enrichment analysis (Fig. 3c).

Using the same $s_{\mathrm{het}}$ estimate, we then calculated the proportion of total selection acting through heterozygotes versus homozygotes for each recessive gene, again under the assumption of recessive lethality. This yields an analytical lower bound on the fraction of selection operating through heterozygotes ($\hat{\;f_{\mathrm{het}}}$; Fig. 3f), as described in the Results.

### Recessive gene QQ plots for quantitative traits

Quantile–quantile plots were generated for all recessive genes using −log10(*p*-values) generated from LoF burden tests for a list of 161 UKB quantitative traits and subset of 21 uncorrelated quantitative traits. Observed −log10(*p*-values) were compared to expected values from a uniform distribution. 95% confidence intervals were generated from a beta distribution using the 2.5% and 97.5% for the *i*th smallest expected *p*-value of the uniform distribution.

### Aggregated Cauchy association tests

For each recessive disease gene, Aggregated Cauchy Association Test (ACAT) was used to combine all LoF burden test *p*-values for the gene (Liu et al. 2019). We performed this analysis on different sets of burden test results: (1) combining LoF burden tests for all 161 UKB quantitative traits, and (2) combining LoF burden tests for 21 uncorrelated quantitative traits. This analysis would identify recessive genes with significant nonrecessive effects using the available trait data. This analysis was repeated for all 18,398 nonrecessive genes independent of our curated recessive disease gene list. With all the nonrecessive genes, ACAT was used to combine all LoF burden test *p*-values for each gene across the LoF burden tests for all 161 UKB quantitative traits and LoF burden tests for 21 uncorrelated quantitative traits.

### Genomic characterization of recessive disease genes

To assess whether recessive disease genes are distinguished from nonrecessive protein-coding genes by their genomic location or functional features, we performed two complementary analyses. In the first, we tested whether the 796 recessive disease genes are nonrandomly distributed across autosomes relative to all protein-coding genes in the analysis set. For each autosome, the expected number of recessive genes was computed under the null hypothesis that recessive genes are distributed proportionally to the total number of protein-coding genes per chromosome. We assessed overall deviation from proportionality using a chi-squared goodness-of-fit test and tested per-chromosome enrichment or depletion using two-sided Fisher’s exact tests with Bonferroni correction for 22 tests. Chromosome assignments for each gene were obtained from the *org.Hs.eg.db* Bioconductor annotation package using Ensembl gene identifiers.

In the second analysis, we compared recessive and nonrecessive genes across a panel of genomic features spanning gene constraint, gene expression, promoter sequence composition, and chromatin environment (Supplemental Table 4). Gene-level total mutation rates and $s_{\mathrm{het}}$ estimates were derived from gnomAD v4.0 as described above. Gene expression metrics were obtained from the GTEx v8 release, which reports median gene-level transcripts per million (TPM) across 54 tissues from 948 postmortem donors (The GTEx Consortium 2020). For each gene, we computed the maximum and mean of the median TPM values across all tissues. GC content was calculated for the promoter region (TSS ±2kb) of each gene from the GRCh37 reference genome sequence. Chromatin environment features were derived from the Roadmap Epigenomics Consortium 15-state ChromHMM model for the E003 reference epigenome (H1 human embryonic stem cells) (Kundaje et al. 2015). Promoter activity was defined as the base-pair-weighted fraction of the TSS ±2kb window annotated as active promoter states (TssA, TssAFlnk). DNase-seq chromatin accessibility was quantified as the mean $-\log_{10}\;(p\text{-value})$ signal from the consolidated DNase-seq track within the same promoter window. Enhancer density was defined as the fraction of the gene body ±10kb annotated as enhancer states (EnhG, Enh). Differences in feature distributions between recessive and nonrecessive genes were assessed using Wilcoxon rank-sum tests. Within recessive genes, we tested Spearman rank correlations between each feature and the estimated proportion of total selection acting through heterozygotes. All *p*-values were Bonferroni-corrected for the number of features tested within each analysis.

### Identifying and analyzing purely recessive genes

Recessive genes that possessed a $p_{\mathrm{LoF}}$ greater than their respective square root total mutation rate were identified to follow classic recessive mutation–selection balance and potentially only be affected by homozygous selection. As population-specific $p_{\mathrm{LoF}}$ were calculated, the list of “purely” recessive genes varied by population and were individually identified.

Gene Ontology (GO) enrichment analysis was performed on recessive genes identified as “purely” recessive in the NFE, FIN, AFR, and SAS populations to identify shared biological, molecular, or cellular function among identified recessive genes (Ashburner et al. 2000; The Gene Ontology Consortium et al. 2023).

### Mutation–selection balance with balancing selection

To understand the potential role of balancing selection on the mutation–selection balance for genes associated with recessive diseases, we visualized the relationship between $p_{\mathrm{LoF}}$ and square root total mutation rate for genes previously suspected to be affected by balancing selection and are associated with only recessive diseases (Clark 1998; Risch et al. 2003; Andrés et al. 2009; Alfonso-Sánchez et al. 2010; Garbade et al. 2019). We performed this analysis using observed $p_{\mathrm{LoF}}$ from NFE, FIN, AFR, and SAS populations available from gnomAD v4.0 genomic information.

## Results

Mutation–selection balance predicts the total allele frequency of all pathogenic variants for a gene, $p_{\mathrm{path}}$, by calculating the frequency at which the rate of new pathogenic alleles arising by mutation is exactly offset by the removal of alleles by natural selection:


(5) $$2\mu L=2p_{\mathrm{path}}(1-p_{\mathrm{path}})s_{\mathrm{het}}+2p_{\mathrm{path}}^{2}s_{\mathrm{hom}},$$

where *μ* denotes the per-base pair mutation rate and *L* is the effective number of sites that can produce pathogenic mutations (Haldane 1927, 1937). When selection on heterozygotes, $s_{\mathrm{het}}$, is non-negligible (i.e. when mediated by dominant or codominant effects on traits), nearly all selection occurs in heterozygotes and the equilibrium frequency can be approximated as


(6) $$p_{\mathrm{path}}=\frac{\mu L}{s_{\mathrm{het}}};\;s_{\mathrm{het}}\gg 0.$$

In contrast, for fully recessive genes, selection occurs only in homozygotes, so the equilibrium frequency is determined entirely by the frequency of homozygotes:


(7) $$p_{\mathrm{path}}=\sqrt{\frac{\mu L}{s_{\mathrm{hom}}}};\;s_{\mathrm{het}}\approx 0.$$

**Fig. 1.**
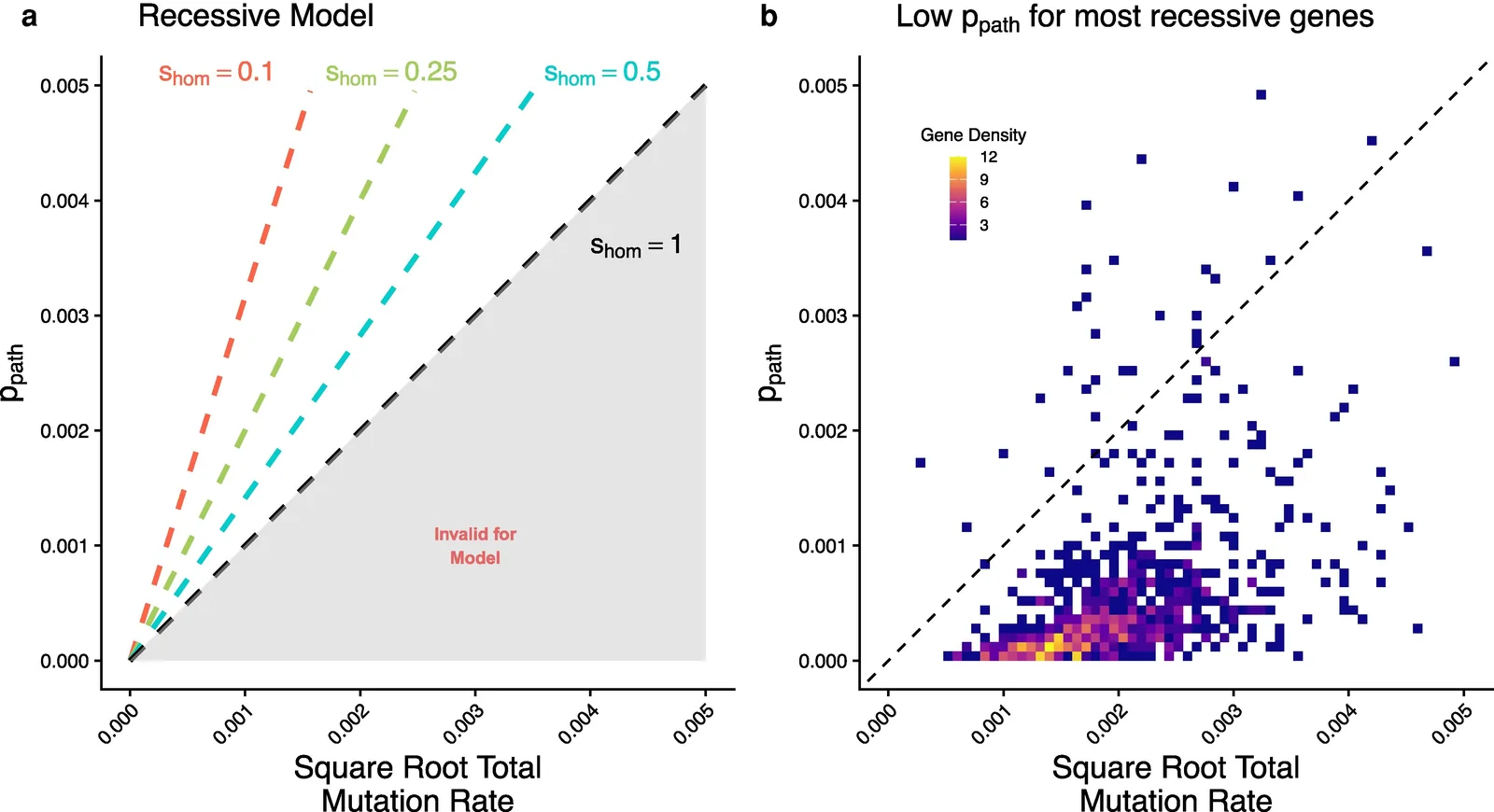
Observed pathogenic allele frequency versus expected: a) theoretical relationship between the square root mutation rate and $p_{\mathrm{path}}$ under recessive mutation–selection balance, for a range of values of homozygous selection, $s_{\mathrm{hom}}$. The shaded area represents parameter combinations that are incompatible with classic recessive mutation–selection balance. b) Density plot showing the observed relationship between square root total mutation rate and predicted $p_{\mathrm{path}}$ for recessive genes. Most genes lie in the parameter region that is inconsistent with classic mutation–selection balance.

Under this model, the frequency of pathogenic alleles, $p_{\mathrm{path}}$, is proportional to the square root of the gene’s total mutation rate μL (Fig. 1a). A key prediction for the present paper is that, even for fully lethal recessive mutations ($s_{\mathrm{hom}}=1$), selection cannot push the equilibrium frequency, $p_{\mathrm{path}}$, below $\sqrt{\mu L}$.

Note that this classic derivation makes several simplifying assumptions that may not hold in practice (i.e. ignoring inbreeding, drift, nonequilibrium population dynamics, and in-phase compound heterozygotes). We will consider these below.

*Curation of recessive disease genes*. To assess the extent to which purely recessive mutation–selection balance pertains to human genes, we curated a list of genes associated with only recessive diseases. To identify the most plausible purely recessive genes, we intersected the 19,194 protein-coding genes defined from the HUGO Gene Nomenclature Committee (Seal et al. 2023), with previous lists of recessive disease genes (Blekhman et al. 2008; Berg et al. 2013; Amorim et al. 2017). We then further filtered to remove genes with nonrecessive effects reported in the OMIM database (Amberger et al. 2015). This removes 387 genes, such as *CFTR* (cystic fibrosis) and *HBB* (sickle cell anemia), that are annotated as recessive, but also have documented heterozygous or nonrecessive effects.

This results in a final list of 796 genes with the strongest support for a fully recessive mode of inheritance (Supplementary Table 1). To assess whether this gene set is genomically representative of protein-coding genes in general, we examined the chromosomal distribution and genomic features of recessive disease genes relative to all nonrecessive protein-coding genes included in the analysis. Recessive disease genes show no significant enrichment or depletion on any autosome relative to expectations based on protein-coding gene density (Supplementary Table 2), and they are indistinguishable from nonrecessive genes with respect to gene expression, chromatin accessibility, promoter activity, enhancer density, and promoter GC content (Supplementary Table 3). The only features that differ significantly between the two sets are total mutation rate and estimated $s_{\mathrm{het}}$. While the $s_{\mathrm{het}}$ result is expected due to previous observations (Zeng et al. 2024) and the given framework employed here, the difference in total mutation rate is unexpected and worthy of further investigation. These results indicate that the deviation from recessive mutation–selection balance observed in subsequent analyses is unlikely to reflect systematic genomic differences between annotated recessive and nonrecessive genes.

*Comparison of $p_{\mathrm{path}}$ to model predictions*. Previous studies of selection coefficients in heterozygotes ($s_{\mathrm{het}}$) have generally focused on estimating the frequencies of certain types of LoF mutations that can be accurately predicted computationally, and for which it is relatively straightforward to model the corresponding mutation rates. Specifically, recent studies have focused on annotating stop-gain, splice site donor-/acceptor-disrupting variants (Cummings et al. 2020; Karczewski et al. 2020).

Here, we refer to these specific categories of mutations as LoF mutations, and we denote the aggregate frequency of LoF variants as $p_{\mathrm{LoF}}$. Gene-level information about LoF variants, including positions and allele frequencies, were retrieved from the gnomAD database v4.0, calculated separately for the NFE, FIN, AFR, and SAS, ancestry groups (Cummings et al. 2020; Karczewski et al. 2020; Stolyarova et al. 2025). We excluded variants with an allele frequency of >0.005 as previous work has shown that these are highly enriched for LoFs that are either bioinformatic errors, or may otherwise be functionally rescued (Zeng et al. 2024). (We show below that our qualitative results hold even without this filtering.)

We estimated the gene level mutation rate $\mu L_{\mathrm{LoF}}$ (to LoFs) for each gene by counting all possible LoF-causing point mutations. We then computed a context-specific mutation rate that accounts for trinucleotide context and methylation status at CpGs for each possible LoF mutation; this was averaged across all *L* possible LoF-generating mutations, to estimate a total gene-level mutation rate (Cummings et al. 2020; Karczewski et al. 2020).

Initial analysis showed that, for nearly all recessive disease genes, the frequency of LoFs, $p_{\mathrm{LoF}}$, lies far below the expected minimum frequency of $\sqrt{\mu L_{\mathrm{LoF}}}$ indicating that $p_{\mathrm{LoF}}$ cannot be predicted by mutation–selection balance under a purely recessive model (Supplementary Fig. 1a). However, this calculation fails to capture the fact that many individuals who are impacted by recessive disease are compound heterozygotes, carrying different mutations on each of their two gene copies. Thus, strictly speaking, the theory only applies to the full set of *all* possible pathogenic mutations, $p_{\mathrm{path}}$.

Compared to annotating LoF mutations, it is much more difficult to confidently identify *all* pathogenic mutations for a given gene, let alone to estimate the total mutation rate that *could* generate pathogenic mutations.

As an alternative, we estimated a scaling factor that represents the fraction of all pathogenic mutations captured by LoFs. Specifically, we identified the molecular consequences of all known pathogenic variants for 25 well-studied recessive disease genes (Supplementary Fig. 1b) and then calculated the percentage of this variation that is captured by our annotated LoF mutations (Supplementary Fig. 1c and Supplementary Table 4). We found that, on average, LoFs contributed around 32% of the total number of pathogenic variants. We then used the inverse of this fraction as a conversion factor to rescale $p_{\mathrm{LoF}}$ and the LoF mutation rate $\mu L_{\mathrm{LoF}}$ to estimate an overall $p_{\mathrm{path}}$ and total mutation rate μL for each gene.

We were intrigued to find that, for the vast majority of recessive disease genes, the estimated $p_{\mathrm{path}}$ is far lower than can be explained by the recessive mutation–selection balance model (Fig. 1b). This result is observed when examining the relationship between $p_{\mathrm{path}}$ and total mutation rate in recessive disease genes in other populations (Supplementary Fig. 2).

When the list of recessive genes is restricted to recessive-lethal genes, which are under the strongest homozygous selection, we also see quantitatively similar results (Supplementary Fig. 3a). This subset represents the most extreme proxy for disease severity available in current gene annotations, and the poor fit of even these genes to the purely recessive model indicates that the deviations we observe are not confined to conditions with modest fitness consequences.

Additional sensitivity analyses were performed to confirm that recessive disease gene selection and variant selection criteria, which might result in underestimating $p_{\mathrm{path}}$, were not likely factors to explain these observations. We tested whether the data might fit if we removed the filter on unusually high-frequency LoFs (MAF >0.005), but this makes little difference to the qualitative results (Supplementary Fig. 3b). We also wondered whether these observations might be explained if we are underestimating the ratio of LoFs to total pathogenic mutations. However, we find that we would need to increase this factor by around 104-fold to make 50 of genes fit the model and need to increase this factor an implausible amount (1,465-fold) to make 90 of genes fit the model.

To better understand why recessive disease genes are largely inconsistent with recessive mutation–selection balance, we next considered two potential categories of explanations: deviations from the basic model including inbreeding and drift, and unexpected quantitative effects in heterozygotes.

### Potential model deviations

The classic mutation–selection balance model makes three important simplifications that may not hold in practice: no inbreeding, no drift, and modeling the gene essentially as a single site with high mutation rate. We consider each of these in turn.

**Fig. 2.**
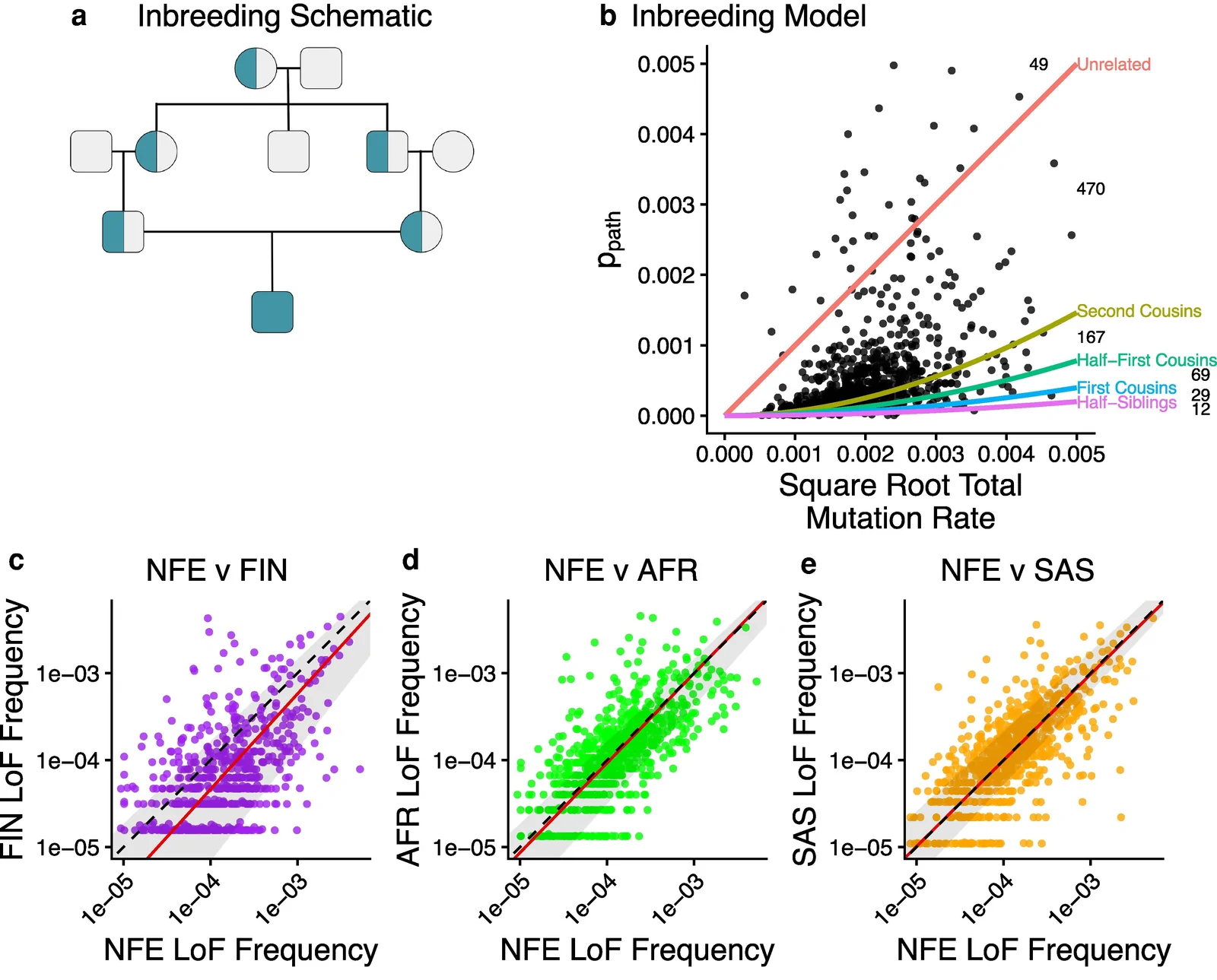
Inbreeding under mutation–selection balance: a) illustration of how inbreeding, in this case between first cousins, increases the frequency of homozygotes in a population (conditional on $p_{\mathrm{path}}$). b) The lines show the theoretical equilibrium $p_{\mathrm{path}}$ under different levels of inbreeding accounting for the potential effects of genetic drift; these are superimposed onto the observed data (e.g. dot is a single recessive gene). The numbers indicate the number of genes that lie between each adjacent pair of theoretical lines. Comparison of $p_{\mathrm{LoF}}$ of recessive genes between NFE and c) FIN populations, d) AFR populations, or e) SAS populations, respectively. Red line represents the total least squares regression of $p_{\mathrm{LoF}}$ between populations. Shaded region represents 95% confidence interval of regression line. The black lines indicate y=x.

*Inbreeding*. In our standard model, the probability that an individual is homozygous for pathogenic mutations follows Hardy-Weinberg expectations, i.e. $p_{\mathrm{path}}^{2}$. However, many populations exhibit some degree of inbreeding—i.e. relatives mate with one another more often than expected under purely random mating. Close inbreeding dramatically increases the probability that an offspring inherits a pathogenic mutation from both parents (Fig. 2a). Thus, conditional on allele frequency, inbreeding increases the probability that pathogenic mutations will be exposed to selection; this results in lower equilibrium frequencies compared to random mating (Charlesworth and Willis 2009). We wanted to assess whether this effect is strong enough to explain our results.

To model this, we measure the extent of inbreeding using the probability that an individual inherits both of their copies of a gene identically by descent, which we denote φ. For example, under random mating φ=0; if 20% of matings in a completely outbred population are between first cousins then φ=0.0125. Under this model, mutation–selection balance takes the following form in the large population size limit:


(8) $$\lim_{N\to \infty }E[p_{\mathrm{path}}]=-\frac{\varphi }{2(1-\varphi )}+\sqrt{{\left(\frac{\varphi }{2(1-\varphi )}\right)}^{2}+\frac{\mu L}{(1-\varphi )s_{\mathrm{hom}}}}$$

See Supplementary Appendix A for details of the model and the derivation of the results.

Equation (8) highlights that higher inbreeding coefficients reduce the expected $p_{\mathrm{path}}$ for a given total mutation rate. However, the extent of inbreeding would need to be implausibly high to explain the data: to explain 95% of recessive genes, we would need a level of inbreeding equivalent to a single generation where *all* matings occur between first cousins (Fig. 2b and Supplementary Fig. 4a).

For comparison, some contemporary inbreeding rates (estimated from 1,000 Genomes data Durbin et al. 2010) identify low levels of second cousin inbreeding in NFE populations and no evidence of first-cousin inbreeding (Gazal et al. 2015) while others (estimated from UK Biobank Data) similarily only identify low rates of second- and first-cousin inbreeding in specific European populations (Yengo et al. 2019). While repeated generations of inbreeding could, in principle, cause identity-by-descent to accumulate beyond the levels implied by a single-generation model, as individuals who already share excess identity-by-descent may preferentially mate within such communities, this effect is unlikely to account for the implausibly high inbreeding coefficients that would be required to fit the observed data. Overall, these results indicate that inbreeding depression is unlikely to explain the failure of recessive mutation–selection balance to fit recessive disease genes.

*Drift in small populations*. Equation (8) assumes an infinite-sized population, neglecting the effects of genetic drift on $p_{\mathrm{path}}$. In contrast to the additive case, genetic drift lowers the expected $p_{\mathrm{path}}$ under mutation–selection balance (Nei 1968; Laws and Jamieson 2011; Hedrick and Garcia-Dorado 2016; Robinson et al. 2018).

To address this, we derived an approximation that quantifies the effects of drift, inbreeding, and selection in homozygotes (Supplementary Appendix A). Our approximation provides a good fit to forward-in-time Wright-Fisher simulations (Supplementary Fig. 4b). Under this adjusted model, inbreeding has a greater effect in reducing $p_{\mathrm{path}}$, which enables more recessive disease genes to fit mutation–selection balance for lower levels of inbreeding. However, even when accounting for drift, fitting the data for most genes would still require inbreeding on the level of ubiquitous first cousin mating (Supplementary Fig. 4c and d). We also argue in Supplementary Appendix A that violations of the assumption that populations are at equilibrium are unlikely to alter these findings.

We also reasoned that if either of these demographic factors (inbreeding or drift) is somehow much larger than we expect, then it would likely vary in magnitude across populations. We would not expect that the precise parameters are the same in diverse populations. But when we analyze the fit for $p_{\mathrm{path}}$ in diverse human populations we find only slight variation among populations and no clear bias (Fig. 2c–e), a result previously described by Stolyarova et al. (2025). While there is evidence of a higher value of φ in SAS populations (Gazal et al. 2015; Wall et al. 2023), this does not result in the systematic reduction of $p_{\mathrm{LoF}}$ relative to populations with lower levels φ that we would expect to see under a purely recessive model. The quantitative similarities among diverse populations suggest that a biological explanation is more likely than a demographic explanation.

*In-phase compound heterozygotes*. Lastly, the standard model does not precisely model compound heterozygotes. These occur when an individual inherits a pair of pathogenic mutations at different sites within the same recessive gene. Typically, a compound heterozygote would have the disease if the two mutations are out-of-phase (i.e. damaging both gene copies), but not if the mutations are in-phase (i.e. damaging only one gene copy). Since selection does not act against in-phase compound heterozygotes, the net effect of this is to weaken selection, and *increase* the expected $p_{\mathrm{path}}$ (Guo et al. 2024; Lassen et al. 2024). Since this effect is in the opposite direction of what we see, we do not consider this scenario further.

### Evidence for heterozygous effects.

We next considered an alternative model in which pathogenic variants within recessive disease genes have effects in heterozygotes that are nonclinical, yet large enough to be relevant for selection. Since heterozygotes are so much more common than homozygotes, even weak selection in heterozygotes may be relevant for determining $p_{\mathrm{path}}$ (Amorim et al. 2017).

**Fig. 3.**
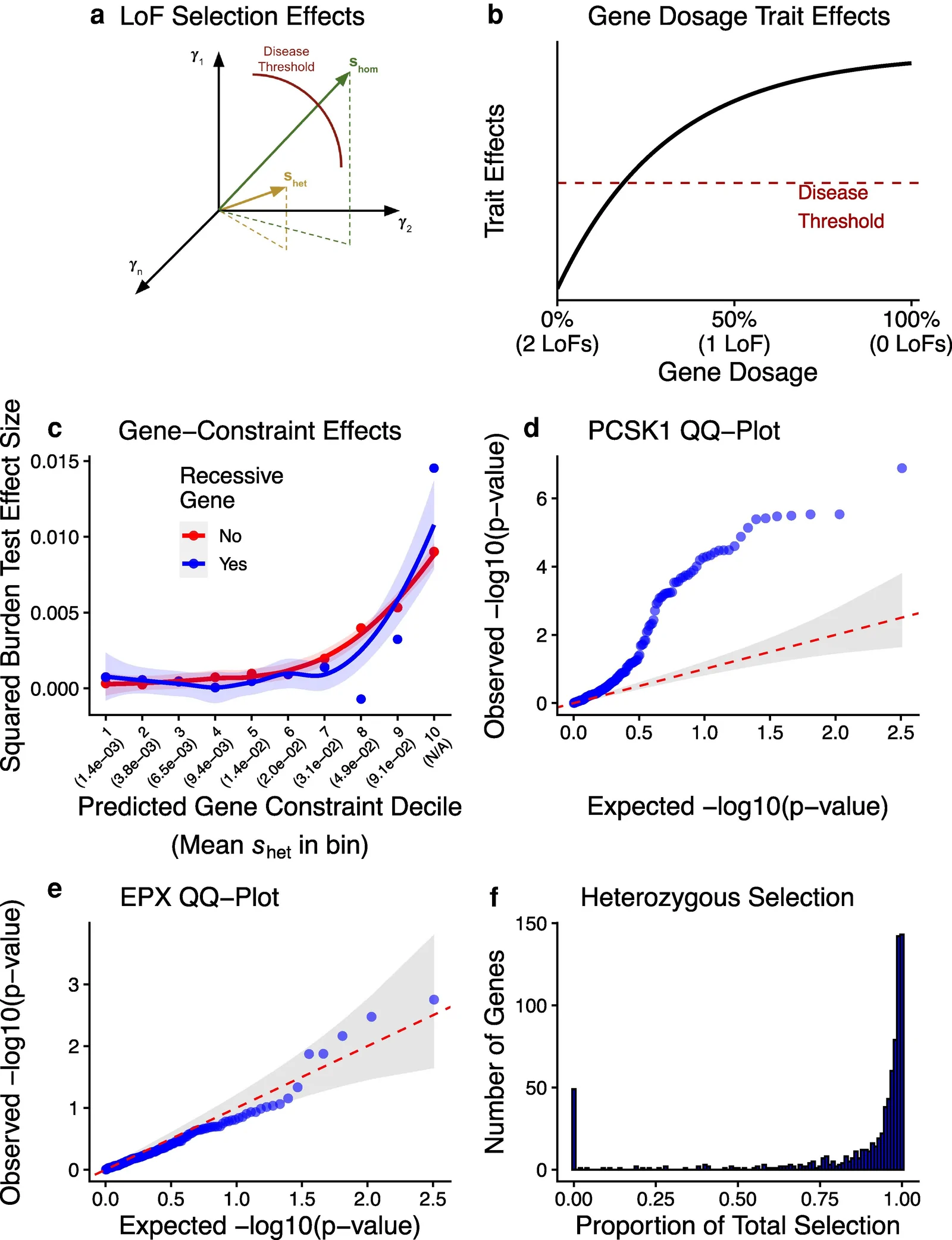
Heterozygous burden effects for recessive genes: a) variant effects can be conceptualized as vectors in a high-dimensional trait space (Simons et al. 2018), shown here for hypothetical heterozygous and homozygous LoFs. Here, only the homozygous LoF crosses a clinical threshold for disease, but both genotypes are subject to selection (selection is proportional to the squared magnitude of the vector). b) Hypothetical relationship between gene expression dosage and model trait effects (Naqvi et al. 2023). The red line represents the trait effect level where a clinical diagnosis would be made. c) Relationship between estimated heterozygous gene constraint decile and average bias-corrected squared LoF burden test effect sizes for 161 quantitative traits, split according to whether or not the genes are classified as recessive. Parentheses denote the mean predicted $s_{\mathrm{het}}$ within each decile bin, calculated across both recessive and nonrecessive genes. The tenth decile is labeled N/A because $s_{\mathrm{het}}$ is indeterminate for recessive genes with $p_{\mathrm{LoF}}=0$ under the recessive-gene constraint estimation method. Lines show loess curve fits and standard errors describing the relationship between constraint and squared burden effect sizes. d) Quantile–quantile plot of 161 LoF burden test *p*-values for the recessive *PCSK1* gene. e) Quantile–quantile plot of 161 LoF burden test *p*-values for the recessive *EPX* gene. f) Histogram showing the numbers of recessive genes with a given proportion of total selection acting through heterozygous LoFs $\left(\hat{\;f_{\mathrm{het}}}\right)$.

Prior work provides evidence that stabilizing selection may be the primary mode of selection for quantitative traits (Simons et al. 2018; Sella and Barton 2019), including for LoFs (Milind et al. 2024; Spence et al. 2025). In this model, the selection against LoF heterozygotes ($s_{\mathrm{het}}$), or other genetic variation, is proportional to a sum of squared effect sizes of that variation on all regulated traits (Fig. 3a) (Simons et al. 2018; Sanjak et al. 2018; Sella and Barton 2019; Simons et al. 2025).

How might this model be relevant to recessive genes? To conceptualize this, we consider the molecular effects of variants that change expression of a relevant gene between 0% (e.g. homozygous LoFs) to 50% (e.g. heterozygous LoFs) to 100% of typical expression. We model these different expression levels as corresponding to different expected values in one of the trait dimensions (Fig. 3b) (Naqvi et al. 2023; Milind et al. 2024; Domingo et al. 2025). We propose that, for recessive disease genes, a single LoF may impact quantitative traits, while being insufficient to cause a clinical disease state (Fig. 3b) (Fridman et al. 2021; Barton et al. 2022; Zschocke et al. 2023; Heyne et al. 2023; Fridman et al. 2025).

To test this model we sought to measure the effects of heterozygous LoFs at recessive genes on quantitative traits measured by the UKB. We performed LoF burden tests on 19,194 genes, including our curated list of 796 recessive disease genes, for 161 quantitative traits. To ensure that we specifically capture the role of heterozygous LoFs on these quantitative traits, we excluded homozygous LoF individuals from the data analysis.

Our group has previously shown that, considering all genes, there is a strong relationship between selective constraint and the average squared effect size of LoFs on quantitative traits (Spence et al. 2025).

For nonrecessive genes, heterozygous gene constraint is estimated using GeneBayes, a Bayesian estimator that provides well-calibrated $s_{\mathrm{het}}$ estimates regardless of observed $p_{\mathrm{LoF}}$ (Zeng et al. 2024); for recessive genes, $s_{\mathrm{het}}$ is instead estimated as a lower bound derived from additive mutation–selection balance applied to the observed $p_{\mathrm{LoF}}$. Because these two constraint measures are derived through methodologically distinct pipelines, the correspondence in burden test enrichment across deciles serves as a consistency check on the core hypothesis: if stabilizing selection on heterozygous trait effects drives constraint in recessive genes, then the mutation–selection balance lower bound on $s_{\mathrm{het}}$ should predict burden enrichment in a manner qualitatively similar to GeneBayes-estimated constraint in nonrecessive genes, as we observe (Fig. 3c).

Were pleiotropic heterozygous effects negligible for recessive genes, the recessive curve would be expected to remain flat across all deciles regardless of constraint level; the close correspondence with the nonrecessive curve instead supports the interpretation that the additive $s_{\mathrm{het}}$ estimates for recessive genes reflects genuine heterozygous fitness consequences. Additionally, a sensitivity analysis in which estimated $s_{\mathrm{het}}$ are scaled downward by up to 75% to account for potential confounding by a combination of inbreeding, drift, and underestimation of $p_{\mathrm{LoF}}$ indicates that the qualitative pattern is robust across this range of assumptions (Supplementary Fig. 5).

In summary, this analysis suggests that widespread heterozygous effects on quantitative traits in recessive disease genes may drive the globally poor fit of the recessive mutation–selection balance model.

*Significance testing of gene–trait pairs*. We also tested the effect of the recessive genes on each of 161 quantitative traits. Although these tests are generally underpowered due to low LoF frequencies and mostly modest effect sizes, we find that 142 of 796 (17.8%) recessive disease genes have significant effects on one or more traits in heterozygotes (Aggregated Cauchy Association Test (ACAT) *p*-value <0.05) (Supplementary Fig. 6a). We find qualitatively similar results when we restrict this analysis to 21 uncorrelated quantitative traits (11.8% of traits significant; Supplementary Fig. 6b). These percentages are similar to what we find for the heterozygous effects of all nonrecessive genes using the same LoF burden test results (14.6 % for all traits; 14.0 % for uncorrelated traits) (Supplementary Fig. 6c and d).

One example of a recessive disease gene with significant heterozygous effects is *PCSK1*, a gene in the same family as *PCSK9* (known for its effects on LDL cholesterol levels (Cariou et al. 2011)). While *PCSK1* is known to cause endocrinopathy in an autosomal recessive fashion (Cool et al. 1997; Jackson et al. 2003), we find that it has effects on BMI, fat accumulation, and other weight-related phenotypes in heterozygotes (Fig. 3d, Supplementary Fig. 6e, and Supplementary Table 5). This indicates that while homozygous LoF of *PCSK1* results in clinical endocrinopathy, variation in *PCSK1* expression such as through heterozygous LoFs have effects on quantitative traits, potentially resulting in selection against such variants (Ramos-Molina et al. 2016; Stijnen et al. 2014, 2016).

In contrast, the recessive gene *EPX* has no observed heterozygous effects and the observed LoF frequency fits recessive mutation–selection balance. *EPX* plays a role in tyrosine nitration granule proteins in resting eosinophils and is recessively linked to eosinophil peroxidase deficiency (Fig. 3e and Supplementary Fig. 6f) (Percopo et al. 2019). While extensive research has identified an association between *EPX* and various phenotypes, such as asthma, allergic response, and airway function, these relationships have primarily been identified in studies with total gene knockouts (Nair et al. 2013; Klonoff-Cohen and Polavarapu 2016; Percopo et al. 2019; Acharya and Ackerman 2014). However, GWAS have identified associations between *EPX* and molecular traits, such as eosinophil and basophil count (Barton et al. 2021), suggesting that even this gene likely has some degree of phenotypic effects at small-effect variants.

*Estimates of the fraction of heterozygous selection*. Lastly, the estimator for $s_{\mathrm{het}}$ derived from additive mutation–selection balance assuming $s_{\mathrm{hom}}=1$ also permits a direct calculation of the minimum fraction of selective pressure acting through heterozygotes for each recessive gene. We sought to estimate, for each recessive gene, the minimum fraction of selective removal of pathogenic mutations that occurs in heterozygotes, which we refer to as $f_{\mathrm{het}}$, and define as


(9) $$f_{\mathrm{het}}=\frac{2p_{\mathrm{path}}(1-p_{\mathrm{path}})s_{\mathrm{het}}}{2p_{\mathrm{path}}(1-p_{\mathrm{path}})s_{\mathrm{het}}+2p_{\mathrm{path}}^{2}s_{\mathrm{hom}}}.$$

Since $f_{\mathrm{het}}$ depends on the unknown values of $s_{\mathrm{het}}$ and $s_{\mathrm{hom}}$, we set


(10) $$\hat{\;f_{\mathrm{het}}}=0,\;\text{if}\;p_{\mathrm{path}}\geq \sqrt{\mu L},$$

which conservatively assumes that the allele frequencies are consistent with fully recessive selection; and otherwise we compute a lower bound on $f_{\mathrm{het}}$ as follows:


(11) $$\hat{\;f_{\mathrm{het}}}\leq 1-\frac{\;p_{\mathrm{path}}^{2}}{\mu L},$$

which makes the conservative assumption that homozygotes have zero fitness, i.e. $s_{\mathrm{hom}}=1$, to maximize the amount of selection occurring in homozygotes (Supplementary Appendix A).

Applying this to empirical data, we find that for most genes, most selection acts through heterozygotes, even when we assume LoFs are homozygous lethal. Out of all recessive genes, only 74 genes (9%) are consistent with being primarily driven by homozygous selection (Fig. 3f). Under the conservative assumption of recessive lethality, we estimate that for 73% of annotated recessive genes, at least 90% of the selection acts through heterozygotes; these estimates represent lower bounds on $s_{\mathrm{het}}$ and are therefore not contingent on precise knowledge of $s_{\mathrm{hom}}$.

### High-frequency recessive genes

**Fig. 4.**
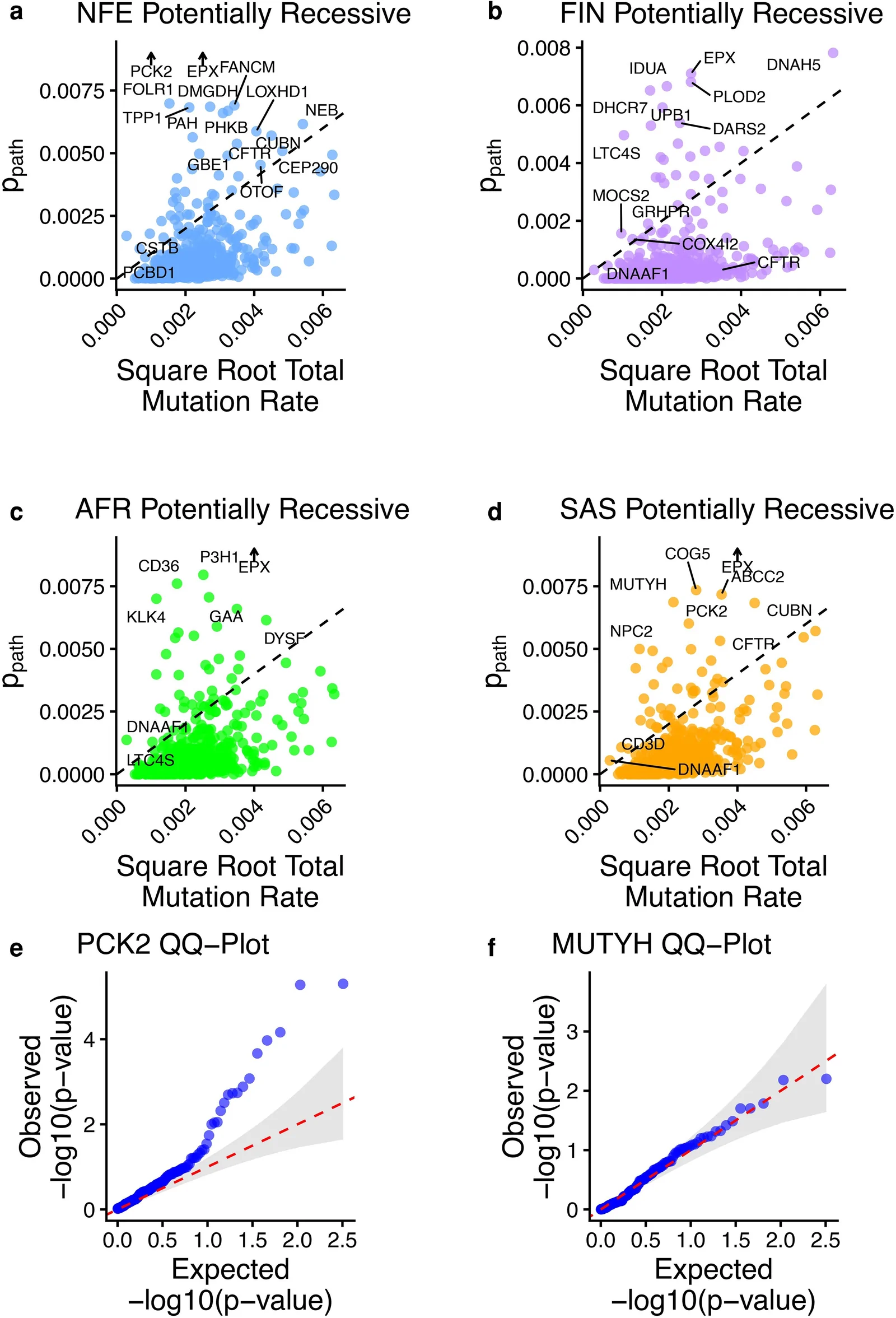
Recessive genes under potentially recessive selection: observed relationship between square root total mutation rate and predicted $p_{\mathrm{path}}$ for recessive disease genes. Chosen recessive disease genes with a predicted $p_{\mathrm{path}}$ in the expected range for recessive mutation–selection balance are labeled using a) NFE allele frequencies, b) FIN European allele frequencies, c) AFR population allele frequencies, and d) SAS population allele frequencies. e) Quantile–quantile plot of 161 LoF burden test *p*-values for the recessive *PCK2* gene. f) Quantile–quantile plot of 161 LoF burden test *p*-values for the recessive *MUTYH* gene.

Although most recessive disease genes do not appear to follow recessive mutation–selection balance, a handful of genes in each population do have predicted pathogenic variation frequencies within the range consistent with the fully recessive model (Fig. 4a). While the specific sets of high-frequency genes vary (Fig. 4b–d and Supplementary Table 6), the absolute numbers are quite similar across populations (49 in NFE, 34 in FIN, 39 in AFR, and 43 in SAS).

Genetic drift, and potential variant ascertainment bias (Stolyarova et al. 2025) may explain the differences between populations, and this stochasticity may result in some genes having higher than expected $p_{\mathrm{path}}$. Alternatively, the difference in which genes are consistent with no selection against heterozygotes may be driven by different selective pressures in different populations, for example due to differing environments. Finally, here we only considered selection *against* LoFs, but these genes with high $p_{\mathrm{path}}$ could also be driven by balancing selection or positive selection (Andrés et al. 2009; Booker et al. 2017).

When we examined in different populations genes previously believed to be affected by balancing selection (Clark 1998; Risch et al. 2003; Andrés et al. 2009; Alfonso-Sánchez et al. 2010; Garbade et al. 2019), we observed variation in the genes that fit recessive mutation–selection balance (Supplementary Fig. 7a–d). For example, two recessive disease genes that fit mutation–selection balance based on the study population and are believed to be affected by balancing selection are *PAH* (Supplementary Fig. 7a) and *CFTR* (Supplementary Fig. 7d), which are the genes associated with causing PKU (Oussalah et al. 2020) and cystic fibrosis (Alfonso-Sánchez et al. 2010), respectively. If these genes and other recessive disease genes are under the influence of balancing selection, their $p_{\mathrm{path}}$ would be higher than expected under a model of purely recessive selection, and potentially look consistent with recessive mutation–selection balance based on the specific population analyzed, due to differences in selective pressures, and choice of variant or LoF inclusion criteria.

Alternatively, this analysis may identify genes where the selection pressures acting on the gene and affecting $p_{\mathrm{path}}$ are unknown. A potential example among these recessive genes is *PCK2*, a mitochondrial isozyme of *PEPCK*, which has a well-known function in gluconeogenesis (Duan 2024). *PCK2* is consistent with having no selection against heterozygotes in both the NFE and SAS populations (Fig. 4a and d), and possesses particularly high $p_{\mathrm{path}}$ in the NFE population (Fig. 4a and Supplementary Table 2). When examining the phenotypic effects of this gene, we observe a wide range of heterozygous effects on the 161 traits we analyzed (Fig. 4e), and other research has found that over-expression of *PCK2* is linked to lung cancer risk, diabetes, and obesity (Xue et al. 2024; Vincent et al. 2015). This suggests that perhaps any selection against *PCK2* in heterozygotes caused by these phenotypic consequences is offset by some other, unknown fitness advantage.

All together, these results indicate that even these genes that are consistent with purely recessive selection may have effects in heterozygotes, and need not follow the textbook definition of recessive selection.

On the other hand, some genes appear to be purely recessive by all measures we examined. For example, *MUTYH* fits recessive mutation–selection balance (Fig. 4d) and has no observed heterozygous effects (Fig. 4f). *MUTYH* has a role in base excision repair and is recessively associated with colorectal adenoma risk (Hemker et al. 2025). Recent research has found that *MUTYH* continues to function in suppressing mutations even in the heterozygous LoF state (Young et al. 2023), and only when two LoFs are present does the rate of *de novo* mutations increase, resulting in increased cancer risk. Overall, it seems plausible that *MUTYH* is an example of a gene governed by recessive mutation–selection balance.

We also examined the subset of recessive genes that match the predictions of purely recessive mutation–selection balance in the NFE, FIN, AFR, and SAS populations (Fig. 4a–d) to identify whether these genes showed any particular patterns. The associated diseases as well as cellular functions of these genes were varied and not limited to a specific biological function or system. However, genes that fit mutation–selection balance in the NFE population are enriched compared to all recessive disease genes for polarized epithelial cellular component GO terms (Supplementary Table 7, *p*-value <0.05), which are involved in transport and environmental sensing and have been associated with a variety of diseases including immunological effects (Wilson 1997; Schleimer and Berdnikovs 2017; Lu et al. 2024), possibly indicating positive or balancing selection driven by host/pathogen competition (von Boehmer 1994; Hedrick 2007; Antonovics et al. 2013; Sironi et al. 2015).

## Discussion

The role of heterozygous and homozygous selection on the equilibrium allele frequency of pathogenic variation in a gene has been understood theoretically for nearly a century (Haldane 1937). Purely recessive mutation–selection balance is a classic result found in textbooks and often taught in introductory population genetics courses (Lewontin 1985; Crow 2017). Meanwhile, the study of Mendelian autosomal recessive genes might lead one to believe that many genes should be governed by purely recessive mutation–selection balance.

Here, we find purely recessive mutation–selection balance is a poor fit for nearly every protein coding gene in the human genome, including recessive disease genes. Our analyses reveal that inbreeding is an unlikely explanation for this phenomenon, and observed $p_{\mathrm{LoF}}$ are instead likely shaped by selection against heterozygotes. We emphasize that the departure from the purely recessive model does not reflect a fundamental limitation of the mutation–selection balance framework, which has been shown to provide reasonable approximations of allele frequencies for genes with well-characterized selective effects (Cassa et al. 2017; Weghorn et al. 2019). Rather, we find that heterozygous LoFs in recessive disease genes have quantitative effects that are similar to genes overall, consistent with prior work demonstrating that isolating recessive selection coefficients from population frequency data is inherently challenging in the presence of substantial heterozygous effects (Balick et al. 2022).

While these effects might not rise to the level of being classified as Mendelian disorders, the substantially larger frequency of individuals with heterozygous LoFs means that even modest selection against heterozygotes may contribute substantially to determining $p_{\mathrm{LoF}}$ in recessive disease genes, and for a meaningful proportion of annotated recessive genes our analyses indicate that heterozygous selection is the dominant force. Our results suggest that recessive disease genes are often highly pleiotropic, with effects even in heterozygotes (Barton et al. 2022; Heyne et al. 2023; Fridman et al. 2025), and hence could play roles in many complex diseases. One possible explanation could be that stabilizing selection on a multidimensional phenotypic space naturally generates partial recessivity for fitness even when mutations act additively on the underlying traits (Manna et al. 2011). As a result, any model of selection in humans should default to assuming that selection occurring in heterozygotes is a major factor.

The broad applicability of purely recessive mutation–selection balance had already been questioned in the pregenomics era, at least in *Drosophila*. By 1941, Dobzhansky and Wright (and later in work with Hovanitz) found that recessive lethal chromosomes in *Drosophila pseudoobscura* were too rare to be explained by recessive mutation selection balance and posited that either selection in heterozygotes or inbreeding were driving the frequencies to be lower (Dobzhansky and Wright 1941; Wright et al. 1942). In 1968, Nei re-examined this question by using the mean and variance of the frequency of recessive lethal chromosomes across different *Drosophila* populations, and came to a similar conclusion (Nei 1968). As summarized in Charlesworth and Charlesworth (2010), later work came to broadly similar conclusions but discounted the effects of inbreeding by estimating heterozygous fitness effects of recessive lethal alleles (Crow 1993; García-Dorado and Caballero 2000).

Another aspect of our study worth considering is that the traits tested for heterozygous effects through burden tests are relatively limited. While these traits were chosen to represent a range of continuous nondisease, biochemistry, and anthropometric traits, they are far from exhaustive. Thus, our results provide a conservative estimate of the role of heterozygous pathogenic variants in recessive disease genes, and inclusion of more uncorrelated traits, particularly molecular traits, should only reveal an even broader range of heterozygous effects of variants in recessive genes (Burton et al. 2009; Smith et al. 2022; Ota et al. 2025).

Furthermore, it is difficult to determine if genes with heterozygous effects are classified as recessive because they are understudied, or if the heterozygous effects are too small to be clinically relevant. For example, recessive genes such as *PCSK1* have strong heterozygous effects that are easily identified through LoF burden tests. While identifying these effects can inform our understanding of a wide range of quantitative traits, classifying these strong effect genes as recessive may simply be due to a lack of research. If these genes were excluded, the mutation–selection balance model may appear more valid in its current form. Inversely, there are a large number of recessive genes where we infer that the majority of selection acts through heterozygotes, but no significant gene-trait effects were identified. This can be partially attributed to the previously mentioned issue of a lack of tested traits or a lack of power, insofar as current datasets may be too small to identify multiple subthreshold effects across traits not captured in the current analysis, which account for the effect of variations in these genes over many generations. Resolving this more conclusively will likely require burden testing against larger panels of molecular and cellular phenotypes, as well as increased sample sizes that provide greater power to detect the modest per-trait effect sizes expected under a model of pleiotropic stabilizing selection. Identifying this second class of genes with known recessive effects and minuscule additive effects is particularly crucial for understanding human genetic variation.

In summary, we have explored the relationship between equilibrium allele frequency and natural selection on complex traits. Our results highlight the potential for widespread effects of heterozygous LoFs, even in recessive disease genes. These insights about recessive genes and natural selection will be informative for various downstream analyses, including the study of the genetic architecture of complex traits and investigations of the selective pressures underlying human genetic variation.

## Supplementary Material

iyag150_Supplementary_Data

iyag150_Peer_Review_History

## Data Availability

UK Biobank data are publicly available by request from https://www.ukbiobank.ac.uk. The research was conducted with approved access to UK Biobank data under application number 52374 (PI: pritchard). Supplemental material available at GENETICS online.
