# Supplementary material for: Allele frequencies at recessive disease genes are mainly determined by pleiotropic effects in heterozygotes
Source: Genetics. 2026 Jun 15;234(1):iyag150. doi: 10.1093/genetics/iyag150 (PMC13535258; doi:10.1093/genetics/iyag150)
Supplement: Supplementary file 1 [file iyag150_peer_review_history.pdf]

GENETICS

Allele Frequencies at Recessive Disease Genes are Mainly Determined by Pleiotropic Effects in Heterozygotes

Jonathan Judd, Jeffrey Spence, Nikhil Milind, Linda Kachuri, John Witte, and Jonathan Pritchard

NOTE: The reviews and decision letters are unedited and appear as submitted by the reviewers.
In extremely rare instances and as determined by a Senior Editor or the EIC, portions of a review may be redacted. If a review is signed, the reviewer has agreed to no longer remain anonymous.
The review history appears in chronological order.

Review Timeline:

Submission Date:	2025-12-11
Editorial Decision:	2026-02-18
Resubmission Received:	2026-05-06
Accepted:	2026-05-23

1st Editorial Decision: February 18, 2026

February 17, 2026

GENETICS-2025-308882
Allele Frequencies at Recessive Disease Genes are Mainly Determined by Pleiotropic Effects in Heterozygotes

Dear Dr. Pritchard:

Two experts in the field have reviewed your manuscript, and I and Nick Barton have read it as well. I am pleased to inform you that, with minor revisions, it is potentially suitable for publication in GENETICS. The feedback is generally positive though the reviewers have comments and concerns that need to be addressed in a revised manuscript. You can read their reviews at the end of this email.

It is most important that you address the following in your resubmission: 1) Both Reviewers 1 and 2 ask for some investigations into the set of recessive genes chosen and whether the signal is in anyway concentrated in a subset; 2) There is a concern about the assumption of equilibrium in human populations in which there has been rapid population growth introducing many new, low-frequency alleles; 3) The connection with the classical Drosophila literature (noted by Rev 1 and the SE) needs to be made, both in the main text and in the theoretical appendix. 4) Also, the additional minor comments of all three reviewers can help make the argument's strengths and weaknesses more clear to readers.

We look forward to receiving your revised manuscript. Please let the editorial office know approximately how long you expect to need for revisions.

Upon resubmission, please include:
1. A clean version of your manuscript;
2. A marked version of your manuscript in which you highlight significant revisions carried out in response to the major points raised by the editor/reviewers (track changes is acceptable if preferred);
3. A detailed response to the editor's/reviewers' comments and to the concerns listed above. Please reference line numbers in this response to aid the editors.

Additionally, please ensure that your resubmission is formatted for GENETICS.
https://academic.oup.com/genetics/pages/general-instructions

Follow this link to submit the revised manuscript: Link Not Available

Sincerely,

John Novembre
Associate Editor
GENETICS

Approved by:
Nick Barton
Senior Editor
GENETICS

Reviewer #1 :

The authors present a straightforward and highly legible investigation into the question of whether human genes with LoF variants implicated solely in recessive disorders fit a model of recessive mutation selection balance. They demonstrate that aggregate LoF variant frequencies are, for the vast majority of "recessive genes", too low to be explained by purely recessive selection. Robustness with respect to genetic drift, inbreeding, and uncertainty in the LoF mutational target is assessed. Heterozygous mutation selection balance as the primary determinant of recessive LoF variant frequencies is concluded. Similar quantitative trait association signals in gene-based burden tests between recessive and non-recessive genes is used to support this point.

I find no issue with the methods or primary conclusions of the manuscript. One can easily imagine that the basic results presented in Figures 1 & 2 could be presented in introductory human genetics textbooks. While there is decent literature on recessive selection in humans I am not aware of this particularly intuitive plot having been previously published. The authors also present several novel theoretical results that will be of interested to readers beyond human genetics, particularly in species with small populations sizes and high inbreeding rates. These appear correct up to my ability to review them.

Statistical genetics results are relatively convincing to the notion that recessive and non-recessive genes have similar distributions of burden test association effects. Distributions in Figure S5 certainly appear similar, but the logic in Figure 3C appear somewhat circular. Genes with high estimated heterozygous constraint would also be those most likely to deviate from the pure recessive model. If lower deciles show little to no burden test enrichment for both gene sets, then it difficult to take this as evidence against the pure recessive model for the majority of annotated recessive genes. Or, if we have already rejected the pure recessive model, it is difficult to take this as evidence that this deviation is driven by a pleiotropic pile of effects roughly captured by burden testing. More simply, because most non-recessive genes don't appear to have a burden enrichment, it seems hard to conclude too much about recessive genes. To the extent that the distributions match up, is it just that the underlying shet distributions are very similar. Having that as an additional supplementary figure would make the argument more convincing.

I also wonder whether it is appropriate to treat all "recessive disease genes" as a single class with respect to fitness effects. Some recessive conditions are plausibly very strongly fitness reducing, while many others are not, and it would be useful to see stratified analyses by some proxy for disease severity (early onset or lethality versus later onset or treatable disease) to check whether the main deviations from the pure recessive model are being driven by a subset. Relatedly, some discussion of demography and non equilibrium regimes would be welcome for genes where selection is weak enough that mutation selection balance may not dominate.

I also worry with the paper is that the conclusion that "most selection experienced by pathogenic mutations in recessive disease genes may be due to stabilizing selection in heterozygotes" may be a bit overstated. In figure Figure 2B most genes fall between the second cousins and unrelated lines, and only ~20% of genes have at least one significant burden association. Why should the reader not interpret this as consistent with the potential for purely recessive fitness effects in a majority of recessive genes due to some mix of light inbreeding, drift, and underestimation of the LoF target? Some discussion of what might resolve this more conclusively would be welcome.

Another piece of discussion which would be welcome, especially for readers of Genetics, would be connections to classical population genetics work on lethal chromosomes. For instance, while the authors make use of the theoretical results in Nei (1968), the scientific and empirical arguments there are also incredibly relevant. Nei's core empirical argument was that, given an assumed input rate of new lethals and the observed frequency of lethal-bearing chromosomes, the observed allelism rate among lethal chromosomes was too low to be consistent with models in which lethals are maintained either by purely recessive mutation selection balance or by widespread overdominance. This is conceptually very close to the analysis presented here.

On the human side of things, the primary difficulty has generally been in detecting any evidence of recessive selection at all against a null of mostly heterozygous selection. Balick et al. (2022), for example. Results presented here are perhaps not surprising in light of that body of work.

Since the authors propose pleiotropic fitness effects as generating the heterozygous selection, it might also be helpful to make reference to theoretical models where dominance and apparent recessivity arise naturally under pleiotropic stabilizing selection on a fitness landscape (e.g. Manna et al. 2011, Genetics).

Minor comments:
Page seven, sentence: "An emerging body of work shows that..."
This is somewhat misleading as written, the only one of the cited studies dealing with LoFs is Spence et al. (2025). The authors should instead phrase this as a reference to their own previous work, rather than an "emerging body of work"

Reviewer #2 :

This paper shows that frequencies of loss of function variants are too low to be explained by the balance of recurrent mutation and recessive-only selection. Based on this observation, the authors ask which of several mechanisms (violations of the mutation-selection null: inbreeding, drift, compound effects, and polygenic selection in heterozygotes) can explain the deficit of LoF variants. They provide evidence that inbreeding and drift are not sufficient to explain the pattern but selection in heterozygotes is. One piece of evidence supporting this observation is a similar relationship between constraint and the effect size of heterozygous LoF variants on ~100s of quantitative traits in the UK Biobank regardless of their annotation as "recessive" or not. Remarkably, estimates of a lower bound on the proportion of selection in heterozygotes show that for the majority of genes with a recessive annotation, selection acts primarily via heterozygotes.

The paper is very clear and provides strong evidence that recessive-only selection is insufficient to explain LoF variant frequencies in human populations, and that polygenicity is a key determinant of recessive mutations experience selection. It will therefore be of considerable interest to a number of communities. (This summary is not to mention the very nice derivation/analysis of the Wright-Fisher diffusion with inbreeding in the supplement!) I have only a handful of comments.

- What does the set of the ~800 genes annotated as recessive look like? Is there evidence that their locations are non-random with respect to their locations on the genome (compared to protein-coding sequence, etc.)? Are genes with especially strong quantitative effects in heterozygotes coincident with, e.g., certain chromatin features? I ask because it seems genomic location, nucleotide content, chromatin accessibility, distance to a TSS, etc. may be useful predictors of the bound on the proportion of total selection in heterozygotes under the gene dosage model. Is this the case?

- Is mutation-selection balance a model we would expect to predict LoF frequencies well, or is a null model which can be used to detect interesting deviations? I was curious about this when reading the passages about the inadequacy of the model because I can think of alternative models which are useful but which rarely inspire efforts to test quantitative predictions (e.g., mutation-mutation balance due in part to the timescale). I certainly think that refinements of the mutation-selection balance model would be useful to develop, but imagine another take-away is that departures from the predicted frequencies says that there is something more interesting going on in a particular region of the genome. I'm curious what the authors think.

- Related to the question of mutation-selection balance as a null model, is there evidence for using *equilibrium* predictions of LoF variant frequencies? I expected the idea that we live in a non-equilibrium world to be among the alternative explanations for a lack of predictive power. I appreciate that for LoF variants, selection is probably quite strong and things equilibrate faster (on a timescale of ~1/sqrt(mu*s) generations?) than under, e.g., mutation-mutation balance. Is this indeed the logic?

Relatedly, I think it would be really interesting if the hypothesis that variant frequencies are due to pleiotropic effects in heterozygotes gave rise to (new) predictions for the *timescale* over which systems approach mutation-selection(-drift) balance, or manner in which they do so. Food for thought...

- I think the caption for Fig 2 should emphasize that the figures provide evidence against the inbreeding *and drift* explanations for lower-than-expected LoF variant freqs.

Reviewer #3 :

Review of the manuscript: "Allele Frequencies at Recessive Disease Genes are Mainly Determined by Pleiotropic Effects in Heterozygotes"

Summary:

Several recent studies demonstrated that pathogenic variants that were traditionally considered purely recessive also have health consequences in heterozygous carriers. Judd et al replicate these findings, and, importantly, place them within the population genetics context of mutation-selection balance. Specifically, they show that the allele frequency of pathogenic variants in known recessive disease genes is much lower than expected under a pure recessive model. Thus, negative selection on heterozygous carriers must have maintained allele frequencies at lower levels. Using mathematical modeling and simulations, they show that the results are robust to drift and inbreeding.

Evaluation:
This is an important and well written manuscript. The results are very useful and provide strong theoretical and empirical support to the paradigm shift under which very few genes are actually purely recessive. The examples of genes showing different patterns (allele frequencies, associations with traits) are also informative. The results have important medical and theoretical implications. The methods and theory are rigorous.

Comments:

I only have minor comments.

• Abstract and last line of page 2: the results are suggested to support stabilizing selection. However, I think the evidence here to specifically support the stabilizing selection model is circumstantial. Or maybe the link can be better delineated.
• Add a color bar to Figure 1B and similar supplementary figures.
• The discussion of the impact of compound heterozygosity is a bit confusing. After all, p_path takes into account all variants in a gene. So I'm not sure what is the concern. Perhaps the concern is that the variant frequencies should not be just added. E.g., for two variants with frequencies p1 and p2, the probability that at least one appears on a single chromosome is p1+p2-p1*p2 (assuming independence). Thus, just using p1+p2 slightly overestimates the frequency that needs to be reached. However, this effect is probably very small, and either way, it is unrelated to compound heterozygosity.
• Page 8, line 20 (Figure 3C). The squared effect vs constraint is the same between recessive and non-recessive genes, but this is only when conditioning on the degree of constraint. Whether recessive genes are more or less constrained than other genes is also a relevant question.
• Page 16, first paragraph. I think the implementation of the correction factor is reasonable. But it's worth noticing that for a random X, E[1/X] != 1/E[X], so the interpretation of the average of the inverse of the percentage of LoF variants can be a bit

tricky.
• Page 17, burden test. Why were variants with MAF>1% filtered?
• Page 17, last paragraph. The procedure to correct effect size estimate for bias is not clear.
• Appendix. I read the entire appendix and I understood most of it. But admittedly some parts were quite difficult to track (see some specific examples below). The general idea was clear, but I think it could also help to have a diagram/illustration that outlines the main steps in the derivation. Otherwise, it is easy to get lost in the technical details and miss the big picture.
• Page 49, first paragraph. I could not understand how the effective coefficients of selection were derived from the last equation on page 48. Can the derivation be shown? Also, are these effective coefficients actually used anywhere downstream?
• Page 49, bottom. I did not fully understand the derivation of the fourth moment, probably because I was not familiar with Young's inequality. Maybe an explanation could be added?
• Page 51, first paragraph. I could not entirely understand the "flux" argument. Is it possible to explain in a bit more detail?
• Figures A1-A3 could be refined a bit. Could you please plot the circles (simulation data) in different colors based on the value of N? And the dashed line is not dashed.
• The technical results were well explained, thank you. Maybe the only point is that in page 59, bottom, I would explain how the asymptotic expressions for the A_k coefficients were derived, and I would mention that the odd terms are not needed (this only appeared later, leaving me initially wondering why they were omitted).

Typos (please do not respond):
• Page 9, line 22, "in may" -> "may".
• Page 10, line 6 from the bottom, "affected" -> "be affected".
• Page 15, line 17, "slice" -> "splice".
• Page 15, line 6 from the bottom, "classed" -> "classes".
• Page 47, line 9, "as a the standard" -> "as the standard".

Associate Editor Comments:

I also wondered about the impact of population growth and whether the influx of new rare variants might lead to the result. For strong s - the equilibrium results should hold quite well even in a moderately changing demography but given large rates of human growth it is worth checking. I also am unsure if the effect doesn't even work in favor of the main argument (in that growth increases the survival probability of alleles and thus one might expect them to exceed what is expected without growth). I'm reminded of theory from Carsten Wiuf and also Slatkin & Ranalla where they studied the branching processes associated with rare variants in the presence of growth and selection in which growth rates end up adding to s in the branching process approximation (Wiuf 2001; Slatkin & Rannalal 1997 - among others around that time). Nick also had a comment that it would be nice to connect to the large literature on Drosophila on this same topic. Overall though this is a very strong first submission and we look forward to seeing the revision.

Senior Editor comments:

This is a nice paper, but it really should connect with the extensive literature on this topic in Drosophila. That the frequency of recessive disease alleles is determined by weak selection against heterozygotes was established for recessive lethals in Drosophila by Wright, Dobzhansky & Hovanitz in 1941. (There are some helpful commentaries on the collaboration between Wright and Dobzhansky in Dobzhansky's collected papers edited by Lewontin et al. (Columbia Univ Press 1981).). This was followed by an extensive literature, which showed how the degree of dominance depended on effect size (reviewed in Charlesworth & Charlesworth Genetica 1998 for example), connecting with current interest in the distribution of fitness effects in heterozygotes (DFE).

I don't think that Wright used his stationary distribution (cf p51) in the analysis - the computations would have been demanding. However, he did argue that selection against heterozygotes could not be distinguished from inbreeding, and estimated a composite parameter (s_het+phi). This argument is not quite the same as yours, though I think they are equivalent for low inbreeding. The relation needs to be discussed. I suspect that the stationary distribution was used to understand the frequency of recessive lethals in later work, but am not sure of this (though see rev 1's comment on Nei 1968)

Response to Reviewers

GENETICS-2025-308882

Allele Frequencies at Recessive Disease Genes are Mainly Determined by Pleiotropic Effects in Heterozygotes

We thank the editors and referees for their careful and constructive engagement with our manuscript. The overall conclusions are qualitatively unchanged; however, we believe that the reviewers' comments have helped us improve the clarity and rigor of the work. The major updates are as follows:

1. ***Genomic characterization of recessive disease genes.*** We performed supplementary analyses comparing recessive and non-recessive protein-coding genes across chromosomal distribution and genomic features, with results reported in Supplemental Tables S2 and S3.

2. ***Non-equilibrium demography.*** We added an appendix section with explicit calculations under various non-equilibrium models (changing selection coefficients and population sizes), suggesting that such effects cannot explain our central results.

3. ***Connection to the classical Drosophila literature.*** We now trace the conceptual history of our central question from Dobzhansky and Wright (1941) to the present day.

4. ***Clarification of analytical assumptions and limitations.*** We clarified the interpretation of Figure 3C, revised the discussion of the identified recessive disease gene burden test associations, and clarified some technical details in our methods.

We have additionally revised several passages throughout to more precisely distinguish empirical findings from theoretical expectations

Reviewer 1

Major Comments

Statistical genetics results are relatively convincing to the notion that recessive and non-recessive genes have similar distributions of burden test association effects. Distributions in Figure S5 certainly appear similar, but the logic in Figure 3C appear somewhat circular. Genes with high estimated heterozygous constraint would also be those most likely to deviate from the pure recessive model. If lower deciles show little to no burden test enrichment for both gene sets, then it is difficult to take this as evidence against the pure recessive model for the majority of annotated recessive genes. Or, if we have already rejected the pure recessive model, it is difficult to take this as evidence that this deviation is driven by a pleiotropic pile of effects roughly captured by burden testing. More simply, because most non-recessive genes don't appear to have a burden enrichment, it seems hard to conclude too much about recessive genes. To the extent that the distributions match up, is it just that the underlying shet distributions are very similar. Having that as an additional supplementary figure would make the argument more convincing.

We thank the reviewer for this careful reading of Figure 3C. We view this figure as providing an independent check on the logic of our paper:

If it is true that most selection on recessive genes occurs in heterozygotes, then we should be able to estimate s_{het} for recessive genes using the standard mutation-selection balance model for (partially) dominant variants. If those estimates are sensible, then we predict that the trait effects in heterozygotes should be similar to those for non-recessive genes with similar s_{het}.

In contrast, if selection and trait effects in heterozgyotes are negligible (for example because the low LOF frequencies are due to demographic effects) then the true s_{het} for recessive genes is ~0, and the estimated s_{het} is meaningless. We'd expect the heterozygous trait effects to be ~0 for all values of estimated s_{het}.

Our results in 3c show that the trait effects for heterozygotes in recessive genes are of similar magnitude to trait effects in non-recessive genes, supporting our main model. But we agree with the reviewer that our logic was unclear in the previous version of the manuscript. Therefore we revised Figure 3C (see below) and modified the text describing Figure 3C to clarify these points:

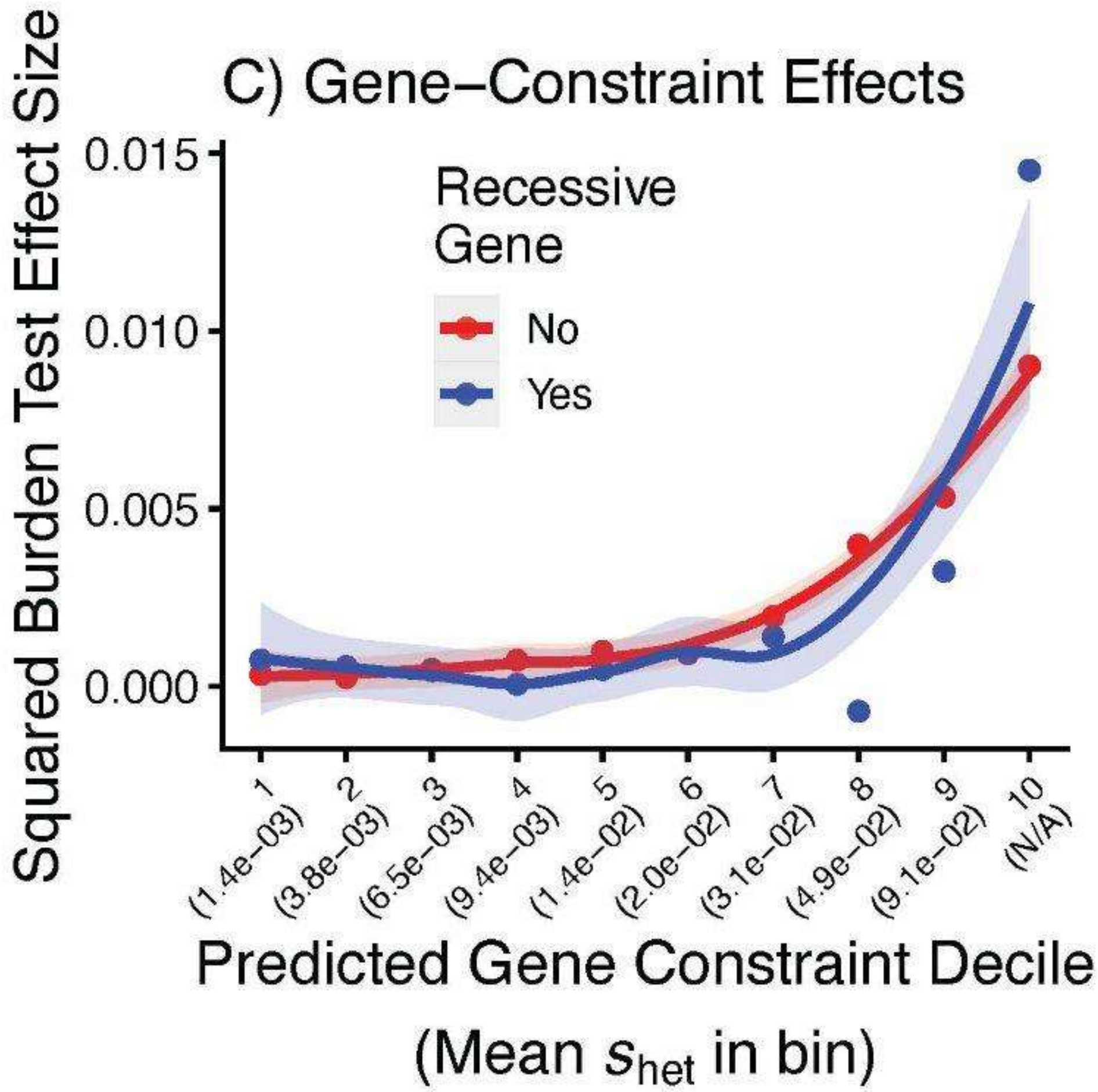

For non-recessive genes, heterozygous gene constraint is estimated using GeneBayes, a Bayesian estimator that provides well-calibrated s_{het} estimates regardless of observed p_{LoF} [10]; for recessive genes, s_{het} is instead estimated as a lower bound derived from additive mutation-selection balance applied to the observed p_{LoF}. Because these two constraint measures are derived through methodologically distinct pipelines, the correspondence in burden test enrichment across deciles serves as a consistency check on the core hypothesis: if stabilizing selection on heterozygous trait effects drives constraint in recessive genes, then the mutation-selection balance lower bound on s_{het} should predict burden enrichment in a manner qualitatively similar to GeneBayes-estimated constraint in non-recessive genes, as we observe (Figure 3C).

Were pleiotropic heterozygous effects negligible for recessive genes, the recessive curve would be expected to remain flat across all deciles regardless of constraint level; the close correspondence with the non-recessive curve instead supports the interpretation that the additive s_{het} estimates for recessive genes reflects genuine heterozygous fitness consequences. Additionally, a sensitivity analysis in which estimated s_{het} are scaled downward by up to 75% to account for potential confounding

by a combination of inbreeding, drift, and underestimation of p_{LoF} indicates that the qualitative pattern is robust across this range of assumptions (Figure S5).

I also wonder whether it is appropriate to treat all "recessive disease genes" as a single class with respect to fitness effects. Some recessive conditions are plausibly very strongly fitness reducing, while many others are not, and it would be useful to see stratified analyses by some proxy for disease severity (early onset or lethality versus later onset or treatable disease) to check whether the main deviations from the pure recessive model are being driven by a subset. Relatedly, some discussion of demography and non-equilibrium regimes would be welcome for genes where selection is weak enough that mutation selection balance may not dominate.

We thank the reviewer for raising both points. Regarding disease severity stratification: we have performed a dedicated analysis restricted to recessive-lethal genes, which represent the strongest possible homozygous selection (shom = 1) and the most extreme proxy for severity available in current annotations. As shown in Figure S3A, even this subset shows p_path values predominantly below the minimum predicted under the purely recessive model, indicating that the deviation is not driven by genes with modest fitness consequences. While these recessive lethal disease genes may fit mutation-selection balance at a higher proportion than the full set of recessive disease genes, finer-grained stratification, such as by age of onset or treatability, would require curation beyond the scope of the current analysis. We add the following in-text when we discuss Figure S3A to clarify this point:

> This subset represents the most extreme proxy for disease severity available in current gene annotations, and the poor fit of even these genes to the purely recessive model indicates that the deviations we observe are not confined to conditions with modest fitness consequences.

Regarding non-equilibrium demography: we made changes to address this directly in Appendix A, where analyses spanning a range of demographic scenarios, (including exponential growth and a demographic model inferred from the 1000 Genomes CEU individuals) indicate that equilibrium predictions provide a reasonable approximation across the relevant range of selection coefficients (Figures A5-A10). Additionally see response to Reviewer 2 for further discussion of non-equilibrium regimes

I also worry with the paper is that the conclusion that "most selection experienced by pathogenic mutations in recessive disease genes may be due to stabilizing selection in heterozygotes" may be a bit overstated. In figure Figure 2B most genes fall between the second cousins and unrelated lines, and only ~20% of genes have at least one significant burden association. Why should the reader not interpret this as consistent with the potential for purely recessive fitness effects in a majority of recessive genes due to some mix of light inbreeding, drift, and underestimation of the LoF target? Some discussion of what might resolve this more conclusively would be welcome.

We agree that the combination of inbreeding, drift, and potential underestimation of LoF targets could in principle reduce the effective s_{het} for recessive genes below our estimates, and that this possibility should be addressed quantitatively. We have conducted a sensitivity analysis in

which the estimated s_{het} values for recessive genes are scaled downward by factors ranging from 0.75 to 0.25, simulating the effect of systematic overestimation. As shown in Supplemental Figure 5, the correspondence between the recessive and non-recessive burden enrichment curves is maintained even when s_{het} estimates are reduced by 25%, and only begins to break down at reductions of 50% or greater as seen by a lack of overlap between 95% confidence interval bands. Achieving such a degree of overestimation simultaneously across the majority of recessive genes would require levels of inbreeding and drift substantially exceeding empirical estimates for non-Finnish European populations (Gazal et al. 2015), making this scenario unlikely.

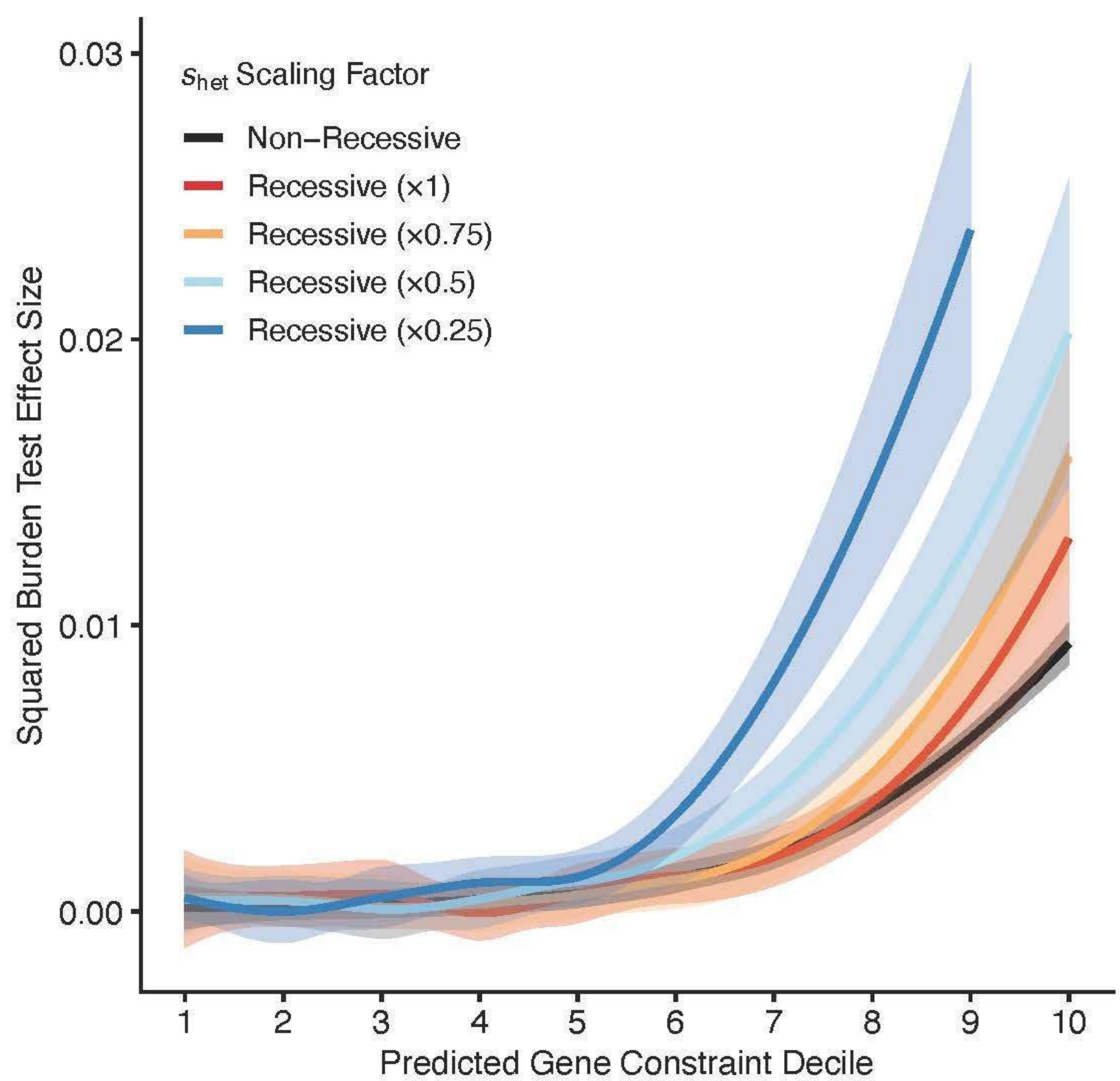

We have nonetheless softened the language in the relevant passages to more accurately reflect the conservative, lower-bound nature of our s_{het} estimates and to acknowledge that, for a subset of recessive genes, a combination of demographic and ascertainment factors may contribute to the observed deviation from the pure recessive model. We note, however, that these considerations do not alter the primary conclusion that for a meaningful proportion of annotated recessive genes, the data are most parsimoniously explained by selection operating through heterozygotes.

Another piece of discussion which would be welcome, especially for readers of Genetics, would be connections to classical population genetics work on lethal chromosomes. For instance, while the authors make use of the theoretical results in Nei (1968), the scientific

and empirical arguments there are also incredibly relevant. Nei's core empirical argument was that, given an assumed input rate of new lethals and the observed frequency of lethal-bearing chromosomes, the observed allelism rate among lethal chromosomes was too low to be consistent with models in which lethals are maintained either by purely recessive mutation selection balance or by widespread overdominance. This is conceptually very close to the analysis presented here.

We thank the reviewer for making this connection to the classic literature. We now include a paragraph tracing our central question all the way back to Dobzhansky and Wright in 1941, including Nei's 1968 work.

> The broad applicability of purely recessive mutation-selection balance had already been questioned in the pre-genomics era, at least in Drosophila. By 1941, Dobzhansky and Wright (and later in work with Hovanitz) found that recessive lethal chromosomes in Drosophila pseudoobscura were too rare to be explained by recessive mutation selection balance and posited that either selection in heterozygotes or inbreeding were driving the frequencies to be lower [81, 82]. In 1968, Nei re-examined this question by using the mean and variance of the frequency of recessive lethal chromosomes across different Drosophila populations, and came to a similar conclusion [83]. As summarized in [84], later work came to broadly similar conclusions, but discounted the effects of inbreeding by estimating heterozygous fitness effects of recessive lethal alleles [85, 86].

On the human side of things, the primary difficulty has generally been in detecting any evidence of recessive selection at all against a null of mostly heterozygous selection. Balick et al. (2022), for example. Results presented here are perhaps not surprising in light of that body of work.

We agree that our findings are broadly consistent with the body of work demonstrating the difficulty of detecting recessive selection signals against a predominantly heterozygous selective background. Balick et al. (2022) demonstrate explicitly that recessive selection coefficients are challenging to recover from population frequency data precisely because the signal is dominated by heterozygous effects, which is consistent with our finding that heterozygous selection appears to govern p_path even in genes annotated as recessive. We have added a sentence to the discussion situating our results within this literature and noting that our approach, which infers the contribution of heterozygous selection indirectly through departure from the recessive model rather than by direct estimation, complements these prior efforts.

> Rather, we find that heterozygous LoFs in recessive disease genes have quantitative effects that are similar to genes overall, consistent with prior work demonstrating that isolating recessive selection coefficients from population frequency data is inherently challenging in the presence of substantial heterozygous effects [15].

Since the authors propose pleiotropic fitness effects as generating the heterozygous selection, it might also be helpful to make reference to theoretical models where dominance and apparent recessivity arise naturally under pleiotropic stabilizing selection on a fitness landscape (e.g. Manna et al. 2011, Genetics).

Thank you for the suggestion. We agree that this work provides a solid theoretical basis to support our central finding. We have added the following in the text in the discussion in regards to this point:

> Our results suggest that recessive disease genes are often highly pleiotropic, with effects even in heterozygotes [21–23], and hence could play roles in many complex diseases. One possible explanation could be that stabilizing selection on a multidimensional phenotypic space naturally generates partial recessivity for fitness even when mutations act additively on the underlying traits [80]. As a result, any model of selection in humans should default to assuming that selection occurring in heterozygotes is a major factor.

Minor Comments

Page seven, sentence: "An emerging body of work shows that..." This is somewhat misleading as written, the only one of the cited studies dealing with LoFs is Spence et al. (2025). The authors should instead phrase this as a reference to their own previous work, rather than an "emerging body of work".

We have clarified that previous work is about the relationship between general *variant* allele frequencies and stabilizing selection.

> "Prior work provides evidence that stabilizing selection may be the primary mode of selection for quantitative traits [40, 41], including for LoFs [20, 42]. In this model, the selection against LoF heterozygotes (s_{het}), or other genetic variation, is proportional to a sum of squared effect sizes of that variation on all regulated traits (Figure 3A) [40, 41, 43, 44]."

Reviewer 2

What does the set of the ~800 genes annotated as recessive look like? Is there evidence that their locations are non-random with respect to their locations on the genome (compared to protein-coding sequence, etc.)? Are genes with especially strong quantitative effects in heterozygotes coincident with, e.g., certain chromatin features? I ask because it seems genomic location, nucleotide content, chromatin accessibility, distance to a TSS, etc. may be useful predictors of the bound on the proportion of total selection in heterozygotes under the gene dosage model. Is this the case?

We have performed two supplementary analyses to characterize the genomic properties of recessive disease genes relative to non-recessive protein-coding genes and to assess whether genomic features predict the proportion of total selection acting through heterozygotes.

In the first analysis (Supplemental Table S2), we tested whether recessive disease genes are non-randomly distributed across autosomes by comparing per-chromosome counts of recessive genes to counts expected under proportional representation relative to all protein-coding genes. After adjusting for multiple testing, we find no significant enrichment or depletion on any chromosome, indicating that recessive disease genes are distributed across the genome in proportion to protein-coding gene density.

In the second analysis (Supplemental Table S3, Panel A), we compared recessive and non-recessive genes across a panel of genomic features spanning gene constraint (s_{het}), gene expression (GTEx v8), promoter sequence composition (GC content), and chromatin environment (ChromHMM promoter activity, DNase-seq accessibility, and enhancer density from Roadmap Epigenomics). After Bonferroni correction, we find that recessive and non-recessive genes are indistinguishable with respect to gene expression levels, chromatin accessibility, promoter activity, enhancer density, and GC content. The only features that differ significantly are total mutation rate and s_{het}. While it has been previously observed that s_{het} in recessive genes is, on average, lower than non-recessive genes (Zeng et al. 2024), the difference in total mutation rate is unexpected and worthy of further investigation.

We also tested whether these genomic features predict the estimated proportion of total selection acting through heterozygotes within recessive genes (Supplemental Table S3, Panel B). Total mutation rate and s_{het} are correlated with this proportion, but both quantities enter directly into its calculation and thus do not represent independent predictive signals. Among the external features, we observe positive correlations with mean gene expression level (Spearman rho = 0.129) and mean enhancer density (rho = 0.136), consistent with the interpretation that more broadly expressed genes embedded in complex regulatory regions may exhibit greater dosage sensitivity. However, chromatin accessibility, promoter activity, and CG content show no significant correlation with the heterozygous selection proportion.

Taken together, these results indicate that recessive disease genes are genomically representative of protein-coding genes in general, and that the deviation from pure recessive mutation-selection balance is not driven by positional biases, regulatory architecture, or

chromatin context. The positive correlations between gene expression, enhancer density, and the proportion of heterozygous selection may reflect a relationship between regulatory complexity and dosage sensitivity, but further investigation would be required to establish a mechanistic basis for this association.

We added the following text in the results section to highlight these results:

> To assess whether this gene set is genomically representative of protein-coding genes in general, we examined the chromosomal distribution and genomic features of recessive disease genes relative to all non-recessive protein-coding genes included in the analysis. Recessive disease genes show no significant enrichment or depletion on any autosome relative to expectations based on protein-coding gene density (Table S2), and they are indistinguishable from non-recessive genes with respect to gene expression, chromatin accessibility, promoter activity, enhancer density, and promoter GC content (Table S3). The only features that differ significantly between the two sets are total mutation rate and estimated s_{het}. While the s_{het} result is expected due to previous observations [10] and the given framework employed here, the difference in total mutation rate is unexpected and worthy of further investigation.These results indicate that the deviation from recessive mutation-selection balance observed in subsequent analyses is unlikely to reflect systematic genomic differences between annotated recessive and non-recessive genes.

Is mutation-selection balance a model we would expect to predict LoF frequencies well, or is a null model which can be used to detect interesting deviations? I was curious about this when reading the passages about the inadequacy of the model because I can think of alternative models which are useful but which rarely inspire efforts to test quantitative predictions (e.g., mutation-mutation balance due in part to the timescale). I certainly think that refinements of the mutation-selection balance model would be useful to develop, but imagine another take-away is that departures from the predicted frequencies says that there is something more interesting going on in a particular region of the genome. I'm curious what the authors think.

We view mutation-selection balance as a model that we would expect to predict LoF frequencies well, and it has previously been shown to provide reasonable approximations of allele frequencies for genes with well-characterized selective effects (Cassa et al. 2017; Weghorn et al. 2019). We are not arguing that the model itself is inadequate, but rather that our understanding of the phenotypic consequences of genes underlying recessive disorders has been incomplete.

This reframing suggests that more rigorous quantitative work is warranted, and that recessive disease genes should be treated as genomically representative of other protein-coding genes in population genetic analyses, rather than characterized solely on the basis of their known homozygous effects, as doing so risks obscuring the broader selective, pleiotropic, and

regulatory forces acting on these loci. We add the following to clarify the fit of the mutation-selection balance model:

> We emphasize that the departure from the purely recessive model does not reflect a fundamental limitation of the mutation-selection balance framework, which has been shown to provide reasonable approximations of allele frequencies for genes with well-characterized selective effects [7, 79]. Rather, we find that heterozygous LoFs in recessive disease genes have quantitative effects that are similar to genes overall.

Related to the question of mutation-selection balance as a null model, is there evidence for using *equilibrium* predictions of LoF variant frequencies? I expected the idea that we live in a non-equilibrium world to be among the alternative explanations for a lack of predictive power. I appreciate that for LoF variants, selection is probably quite strong and things equilibrate faster (on a timescale of ~1/sqrt(mu*s) generations?) than under, e.g., mutation-mutation balance. Is this indeed the logic? Relatedly, I think it would be really interesting if the hypothesis that variant frequencies are due to pleiotropic effects in heterozygotes gave rise to (new) predictions for the *timescale* over which systems approach mutation-selection(-drift) balance, or manner in which they do so. Food for thought...

This is a very interesting point. We have added a section to the Appendix detailing the effects of non-equilibrium dynamics. Equilibration in the purely recessive case is slower than in the case of selection acting on heterozygotes. For an aggregate LoF mutation rate of 10^{-5}, equilibration of recessive lethal alleles takes hundreds of generations in small populations (N=50), and thousands of generations in larger populations (N=50,000). This represents the relevant timescale were an allele to suddenly become recessive lethal. That is, if a neutral allele drifted to some intermediate frequency, it could require thousands of generations to reach mutation selection balance following a switch to being recessive lethal. Many recessive disease genes cause severe phenotypes by disrupting key biological processes, so we suspect that even thousands of generations ago, such genes would still reduce fitness. On the other hand, for populations already at equilibrium, changes in population size have less dramatic effects. Based on a model of recent exponential growth (Tennessen *et al.* 2012), we considered the effects of 176 generations of exponential growth at a rate of 1.95% per generation, and found that the expected frequency of purely recessive variants was largely the same as it would have been at equilibrium in the population prior to exponential growth. We also computed the expected frequency of homozygous lethal alleles under a realistic demography estimated using individuals labeled as CEU in the 1000 Genomes project (Schiffels and Durbin 2014) and found that the results were similar to the expected frequency of a homozygous lethal allele in an equilibrium population of size ~20,000.

Comparing the rates of equilibration of variants exposed to selection only in homozygotes to those with additional effects in heterozygotes would indeed be interesting. Stolyarova, Coop, and Przeworksi (2025) argue that strongly selected LoFs are generally extremely young (e.g., they say that an LoF with a fitness consequence of 5% in heterozygotes is a usually about 5

generations old), which suggests equilibration could be much faster, but would presumably depend on the strength of s_{het}. We leave a careful comparison of these timescales and their implications for future work.

I think the caption for Fig 2 should emphasize that the figures provide evidence against the inbreeding *and drift* explanations for lower-than-expected LoF variant freqs.

The caption for Figure 2 has been revised to emphasize that the analyses provide evidence against both the inbreeding and drift explanations for the lower-than-expected LoF variant frequencies observed in recessive disease genes.

Reviewer 3
Comments
Abstract and last line of page 2: the results are suggested to support stabilizing selection. However, I think the evidence here to specifically support the stabilizing selection model is circumstantial. Or maybe the link can be better delineated.

We thank the reviewer for this point. Our interpretation of the literature is that most of these selective effects are likely due to stabilizing selection, and this provides the framing for our analysis in Figure 3. But we concede that our analysis in this paper does not show this precise point and we have removed the word 'stabilizing' from the Abstract and from Page 2.

Add a color bar to Figure 1B and similar supplementary figures.

Thank you for the comment. Color bars indicating gene density have been added to Figure 1B and Figure S1A.

The discussion of the impact of compound heterozygosity is a bit confusing. After all, p_path takes into account all variants in a gene. So I'm not sure what is the concern. Perhaps the concern is that the variant frequencies should not be just added. E.g., for two variants with frequencies p1 and p2, the probability that at least one appears on a single chromosome is p1+p2-p1*p2 (assuming independence). Thus, just using p1+p2 slightly overestimates the frequency that needs to be reached. However, this effect is probably very small, and either way, it is unrelated to compound heterozygosity.

We agree with the reviewer that this effect should be small in practice, but we brought it up both because there is some confusion in the literature, and because we have observed some confusion in conversations with colleagues. We have tried to slightly improve the wording in the main text but we are reluctant to expand this paragraph since the predicted effect is in the wrong direction and the entire discussion seems a red herring. (To clarify one point, we view this as an issue of compound heterozygotes because all individuals with 2 mutations at the same site are affected, but only ~½ of compound heterozygotes are affected.)

Page 8, line 20 (Figure 3C). The squared effect vs constraint is the same between recessive and non-recessive genes, but this is only when conditioning on the degree of constraint. Whether recessive genes are more or less constrained than other genes is also a relevant question.

We agree that the degree of heterozygous constraint in recessive versus non-recessive genes is a relevant question. As shown in Figure 5A of Zeng et al. 2024, the distribution of GeneBayes-estimated s_{het} values for manually curated recessive genes is broad, spanning from weak to strong selection, and overlaps substantially with distributions observed for other gene sets. This observation, taken together with the correspondence in burden test enrichment across constraint deciles shown in Figure 3C, supports the interpretation that recessive disease genes are not categorically distinct from other protein-coding genes with respect to their heterozygous fitness consequences. The annotation of a gene as recessive reflects the phenotypic manifestation of its homozygous effects, but does not appear to predict meaningful differences in the degree to which heterozygous LoFs influence quantitative traits or the population genetic forces shaping allele frequency distributions at these loci.

Page 16, first paragraph. I think the implementation of the correction factor is reasonable. But it's worth noticing that for a random X, E[1/X] != 1/E[X], so the interpretation of the average of the inverse of the percentage of LoF variants can be a bit tricky.

Thank you for the observation. We agree that the mean of the per-gene inverse LoF fractions is not, in general, equal to the inverse of the mean LoF fraction. Our use of the mean of the per-gene inverses produces a larger correction factor than the alternative approach of averaging the LoF fractions prior to inversion, yielding a more conservative estimate of p_path that provides the purely recessive mutation-selection balance model a greater opportunity to fit the observed data. The fact that this more conservative metric nevertheless reveals a poor fit for the recessive mutation-selection balance model across nearly all recessive disease genes (Figure 1B) further bolsters the robustness of our central conclusions.

Page 17, burden test. Why were variants with MAF>1% filtered?

LoF variants with MAF greater than or equal to 1% were excluded to avoid the inclusion of variants that may tag common variant signal through linkage disequilibrium (LD), which could bias estimated burden test effect sizes upward or downward. Restricting to rare variants with MAF below 1% substantially reduces this concern, as rare variants generally exhibit negligible LD with common variants (Good 2022). This filtering approach is consistent with standard practice in gene-level burden testing analyses (Backman et al. 2021). Additionally, in previous work we have found that LoF variants with MAF greater than 1% tend to be bioinformatics artifacts and misannotated rather than true LoFs (Zeng et al. 2024).

We added a comment to this effect.

Page 17, last paragraph. The procedure to correct effect size estimate for bias is not clear.

We have clarified the protocol for calculating the bias-corrected squared effect sizes in this section.

Appendix. I read the entire appendix and I understood most of it. But admittedly some parts were quite difficult to track (see some specific examples below). The general idea was clear, but I think it could also help to have a diagram/illustration that outlines the main steps in the derivation. Otherwise, it is easy to get lost in the technical details and miss the big picture.
Thank you for carefully reading the mathematical details. We have added a flowchart to the beginning of the Appendix outlining the main steps in our argument to give readers a sense of where everything is going and how each piece fits into the larger argument.

Page 49, first paragraph. I could not understand how the effective coefficients of selection were derived from the last equation on page 48. Can the derivation be shown? Also, are these effective coefficients actually used anywhere downstream?
Thank you for pointing out that this was unclear. The derivation follows by matching the known form from the case with no inbreeding, which was why we had written the expected frequency change in a somewhat strange form. We have tried to make that connection clearer along with an example reference. We do not use these effective coefficients anywhere and include them simply because models without inbreeding are more standard than models with inbreeding, and so we believe that the connection is of independent interest. We have clarified this in the text.

Page 49, bottom. I did not fully understand the derivation of the fourth moment, probably because I was not familiar with Young's inequality. Maybe an explanation could be added?
We have now provided a direct proof of the inequality along with additional explanatory text.

Page 51, first paragraph. I could not entirely understand the "flux" argument. Is it possible to explain in a bit more detail?
We have added some more detail to the flux argument in the hopes of making it clearer.

Figures A1-A3 could be refined a bit. Could you please plot the circles (simulation data) in different colors based on the value of N? And the dashed line is not dashed.

We believe that this may have been an issue on the part of the reviewer's PDF renderer, as these figures already color points based on N and have solid, dashed, and dotted lines.

The technical results were well explained, thank you. Maybe the only point is that in page 59, bottom, I would explain how the asymptotic expressions for the A_k coefficients were

derived, and I would mention that the odd terms are not needed (this only appeared later, leaving me initially wondering why they were omitted).

Thank you for pointing out that we were sweeping too many details under the rug. We have considerably expanded this section of the proof to clarify the argument. Note that we now split the argument into three steps, so what was previously A_k is now denoted as C_k in the revised version.

Associate Editor Comments

I also wondered about the impact of population growth and whether the influx of new rare variants might lead to the result. For strong s - the equilibrium results should hold quite well even in a moderately changing demography but given large rates of human growth it is worth checking. I also am unsure if the effect doesn't even work in favor of the main argument (in that growth increases the survival probability of alleles and thus one might expect them to exceed what is expected without growth). I'm reminded of theory from Carsten Wiuf and also Slatkin & Ranalla where they studied the branching processes associated with rare variants in the presence of growth and selection in which growth rates end up adding to s in the branching process approximation (Wiuf 2001; Slatkin & Rannalal 1997 - among others around that time).

Thank you for pointing us to the branching process papers. We've now included some explicit calculations under an exponential growth model in the Appendix, and discussed how it impacts the results. Our overall finding is that short periods of exponential growth do no largely impact the expected allele frequency, but in response to the comments of Reviewer 1, we explored the impact of non-equilibrium demography and the timescale of equilibration more thoroughly.

The impact of growth in our case is somewhat different from in the branching process literature. In those papers, the distribution of the genealogy of a sample of individuals carrying a particular allele is governed by a compound parameter r + s, indicating that exponential growth should cause the genealogy to look as if it came from a sample of individuals with a more positively selected allele. There are a few differences with our work – first, in the branching process regime, the variant needs to be so rare that homozygotes can be neglected, and so their "s" is our "s_{het}". Second, they ignore the effects of recurrent mutation. Ignoring back mutation is probably fine as the number of individuals with an LoF is small, but recurrence is a powerful force for driving the overall frequency of LoFs. A typical aggregate LoF mutation rate for a gene is ~10^{-5}, which even in a population with an N_e of 20,000 would result in a new LoF appearing on average every 5 generations. Third, the branching process result is purely about genealogical properties, and does not imply that the distribution of present-day frequencies is governed by r + s_{het}. If one considers the SDE governing the allele frequency in an exponentially growing population, it's clear that the effect of growth is to reduce the strength of drift (relative to the effect of drift in the population before growth), but has no effect on the deterministic part of the SDE.

Nick also had a comment that it would be nice to connect to the large literature on Drosophila on this same topic.

We address this below in our response to Nick.

Senior Editor Comments
This is a nice paper, but it really should connect with the extensive literature on this topic in Drosophila. That the frequency of recessive disease alleles is determined by weak selection against heterozygotes was established for recessive lethals in Drosophila by Wright, Dobzhansky & Hovanitz in 1941. (There are some helpful commentaries on the collaboration between Wright and Dobzhansky in Dobzhansky's collected papers edited by Lewontin et al. (Columbia Univ Press 1981).). This was followed by an extensive literature, which showed how the degree of dominance depended on effect size (reviewed in Charlesworth & Charlesworth Genetica 1998 for example), connecting with current interest in the distribution of fitness effects in heterozygotes (DFE).

I don't think that Wright used his stationary distribution (cf p51) in the analysis - the computations would have been demanding. However, he did argue that selection against heterozygotes could not be distinguished from inbreeding, and estimated a composite parameter (s_het+phi). This argument is not quite the same as yours, though I think they are equivalent for low inbreeding. The relation needs to be discussed. I suspect that the stationary distribution was used to understand the frequency of recessive lethals in later work, but am not sure of this (though see rev 1's comment on Nei 1968).

Thank you for pointing out this relevant work. We've added a paragraph to the discussion citing these papers and discussing more of the history (starting with Dobzhansky and Wright, including work by Nei, and continuing to more recently).

> The broad applicability of purely recessive mutation-selection balance had already been questioned in the pre-genomics era, at least in Drosophila. By 1941, Dobzhansky and Wright (and later in work with Hovanitz) found that recessive lethal chromosomes in Drosophila pseudoobscura were too rare to be explained by recessive mutation selection balance and posited that either selection in heterozygotes or inbreeding were driving the frequencies to be lower [81, 82]. In 1968, Nei re-examined this question by using the mean and variance of the frequency of recessive lethal chromosomes across different Drosophila populations, and came to a similar conclusion [83]. As summarized in [84], later work came to broadly similar conclusions, but discounted the effects of inbreeding by estimating heterozygous fitness effects of recessive lethal alleles [85, 86].

We have also added some clarifying remarks to the Appendix, with pointers to relevant work by Wright.

References

1. S. Gazal, M. Sahbatou, M.-C. Babron, E. Génin, and A.-L. Leutenegger, “High level of inbreeding in final phase of 1000 Genomes Project,” Sci Rep, vol. 5, no. 1, p. 17453, Dec. 2015, doi: 10.1038/srep17453.
2. C. A. Cassa et al., “Estimating the selective effects of heterozygous protein-truncating variants from human exome data,” Nat Genet, vol. 49, no. 5, pp. 806–810, May 2017, doi: 10.1038/ng.3831.
3. D. Weghorn et al., “Applicability of the Mutation–Selection Balance Model to Population Genetics of Heterozygous Protein-Truncating Variants in Humans,” Mol Biol Evol, vol. 36, no. 8, pp. 1701–1710, Aug. 2019, doi: 10.1093/molbev/msz092.
4. E. M. Koch and S. R. Sunyaev, “Maintenance of Complex Trait Variation: Classic Theory and Modern Data,” Front. Genet., vol. 12, Nov. 2021, doi: 10.3389/fgene.2021.763363.
5. B. H. Good, “Linkage disequilibrium between rare mutations”, Accessed: Feb. 25, 2026. [Online]. Available: https://dx.doi.org/10.1093/genetics/iyac004
6. J. D. Backman et al., “Exome sequencing and analysis of 454,787 UK Biobank participants,” Nature, vol. 599, no. 7886, pp. 628–634, Nov. 2021, doi: 10.1038/s41586-021-04103-z.
7. T. Zeng, J. P. Spence, H. Mostafavi, and J. K. Pritchard, “Bayesian estimation of gene constraint from an evolutionary model with gene features,” Nat Genet, pp. 1–12, Jul. 2024, doi: 10.1038/s41588-024-01820-9.

May 22, 2026
RE: GENETICS-2026-309463

Dr. Jonathan K. Pritchard
Stanford University
Genetics
Department of Genetics, Mail Stop-5120
Stanford 94305-5120

Dear Dr. Pritchard:

Congratulations, your manuscript titled "Allele Frequencies at Recessive Disease Genes are Mainly Determined by Pleiotropic Effects in Heterozygotes" is accepted for publication in GENETICS! Many thanks for submitting your research to the journal.

Reviewer 2 has one minor suggestions for improving the manuscript that you may want to consider. You can view their comments at the bottom of this email.

To Proceed to Publication:
1. Format your article according to GENETICS style: https://academic.oup.com/genetics/pages/author-guidelines

2. Ensure that you comply with data and community resource citation guidelines:
https://academic.oup.com/genetics/pages/author-guidelines#section-5-9-2

3. Upload your final files at https://genetics.msubmit.net

4. Add oupsupport@scipris.com and genetics.oup@novatechset.com (or the domains @scipris.com and @novatechset.com) to your email program's "safe senders" list. You will be contacted by both at various points during the production process.

Notes:
- Your currently-accepted manuscript (unedited, as submitted, reviewed, and accepted) will be published at GENETICS and deposited into PubMed as an Advance Access article. Notify sourcefiles@thegsajournals.org before signing your license if you do not wish to publish your article via Advance Access.

- We invite you to submit an original color figure related to your paper for consideration as cover art. Please note that submissions created with AI image generation tools are not eligible. Please email your submission to the editorial office or upload it with your final files. You can submit a small-sized image for evaluation, and if selected, the final image must be a TIFF file 2513px wide by 3263px high (8.375 by 10.875 inches; resolution of 600ppi). Please avoid graphs and small type.

- After files are sent to Oxford University Press we use SciPris to manage article licensing and payment. If you do not have a SciPris account, you will receive an email from no-reply@scipris.com to sign up to use Oxford University Press' author portal. After logging in, follow the online instructions to sign your license and arrange any payment due.

If you have any questions or encounter any problems while uploading your accepted manuscript files, please email the editorial office at sourcefiles@thegsajournals.org.

Sincerely,

John Novembre
Associate Editor
GENETICS

Approved by:
Nick Barton
Senior Editor
GENETICS

Review comments (if applicable):

Reviewer #1 :

I am satisfied with the authors' revisions and responses. The manuscript is meaningfully more clear, and I have no additional concerns.

Reviewer #2 :

I greatly enjoyed reading the original manuscript and think that the revision is even better! The authors have addressed all of my comments and I think the additions offer compelling additional support for the idea that frequencies of LoF mutations reflect pleiotropic effects of mutations in heterozygotes.

One suggestion:

I think the description of the genomic characterization of the recessive disease genes in the second paragraph of "Curation of recessive disease genes" might make more sense in another section ("Evidence for heterozygous effects" or the Discussion). It may be confusing for readers to read about the difference in s_het values for the recessive vs. non-recessive genes before the comparison of the mutation-selection balance model and empirical LoF frequencies (and estimation of the s_het lower bound).

Reviewer #3 :

I have no further comments.
