# Supplementary material for: Allele frequencies at recessive disease genes are mainly determined by pleiotropic effects in heterozygotes
Source: Genetics. 2026 Jun 15;234(1):iyag150. doi: 10.1093/genetics/iyag150 (PMC13535258; doi:10.1093/genetics/iyag150)
Supplement: Supplementary file 2 [file iyag150_supplementary_data.zip › Supplemental_Figures_&_Tables_GENETICS-2026-309463.pdf]

Supplemental information

Contents

List of Supplemental Figures 35

List of Supplemental Tables 44

List of Supplemental Figures

Supplemental Figure 1:
a) Density plot for observed relationship between square root mutational target size and p_{LoF} for recessive genes using NFE allele frequencies. **b)** Proportional split of observed pathogenic variation for 25 commonly-studied recessive disease genes based on predicted molecular consequence (LoF, Frameshift, Missense, or Other). **c)** Percentage of observed pathogenic variation for 25 commonly-studied recessive disease genes predicted to be LoFs. Bands represent two standard error confidence intervals surrounding LoF percentage point estimate. Red line represents average percentage of pathogenic variation predicted to be LoFs between all recessive disease genes. 37

Supplemental Figure 2:
a) Observed relationship between square root mutational target and p_{path} for recessive genes using NFE allele frequencies. **b)** Observed relationship between square root mutational target and p_{path} for recessive genes using FIN allele frequencies. **c)** Observed relationship between square root mutational target and p_{path} for recessive genes using AFR allele frequencies. **d)** Observed relationship between square root mutational target and p_{path} for recessive genes using SAS allele frequencies. 38

Supplemental Figure 3:
a) Observed relationship between square root mutational target size and p_{path} for recessive lethal disease genes. Genes with a predicted p_{path} in the expected range for recessive mutation-selection balance are labeled using non-Finnish European (NFE) allele frequencies. **b)** Observed relationship between square root mutation target size and p_{path} for recessive genes using NFE allele frequencies and not excluding LoF variants with a frequency greater than 0.005 prior to calculating p_{path}. 39

Supplemental Figure 4:
a) Percentage of recessive disease genes that fit the classic recessive mutation-selection balance for different levels of population inbreeding. **b)** Comparison of the classic recessive mutation-selection balance model and adjusted Nei model (Equation 9) for a finite population for populations with no inbreeding. Lines represent predicted p_{path} for a given mutational target size for each model assuming recessive lethality. Dots represent simulated p_{path} data for recessive lethal genes with varying mutational target sizes. **c)** Observed relationship between square root mutational target size and p_{path} for recessive genes using NFE allele frequencies. Lines represent the expected relationship between square root mutational target size and p_{path} under finite population model for different levels of inbreeding in the population. **d)** Percentage of recessive disease genes that fit the finite population model for different levels of population inbreeding. 40

Supplemental Figure 5:
Relationship between predicted gene constraint decile and average bias-corrected squared LoF burden test effect sizes for 161 quantitative traits. Recessive gene s_{het} values are scaled by factors of $\times 1$, $\times 0.75$, $\times 0.5$, and $\times 0.25$ prior to joint ranking with non-recessive genes into constraint deciles. The non-recessive reference curve (black) is held fixed across all scaling scenarios. Analysis is restricted to the top 75% of genes by mutational target size. Lines show loess curve fits and shaded bands represent 95% confidence intervals. 41
Supplemental Figure 6:
a) Empirical cumulative distribution function of ACAT omnibus test of p-values from LoF burden tests for 161 UK BioBank (UKB) quantitative traits across 796 recessive disease genes. The vertical line represents p-values that surpass 0.05. **b)** Empirical cumulative distribution function of ACAT omnibus test of p-values from LoF burden tests for 21 uncorrelated UKB quantitative traits across 796 recessive disease genes. The vertical line represents p-values that surpass 0.05. **c)** Empirical cumulative distribution function of ACAT omnibus test of p-values from LoF burden tests for 161 UK BioBank (UKB) quantitative traits across a random sample of 796 non-recessive disease genes. The vertical line represents p-values that surpass 0.05. **d)** Empirical cumulative distribution function of ACAT omnibus test of p-values from LoF burden tests for 21 uncorrelated UKB quantitative traits across a random sample of 796 non-recessive disease genes. The vertical line represents p-values that surpass 0.05. **e)** Quantile-quantile plot of 21 LoF burden test p-values from uncorrelated traits for the recessive *PCSK1* gene. **f)** Quantile-quantile plot of 21 LoF burden test p-values from uncorrelated traits for the recessive *EPX* gene. 42
Supplemental Figure 7:
a) Observed relationship between square root mutational target and p_{path} for recessive disease genes previously thought to be affected by balancing selection using NFE allele frequencies. **b)** Observed relationship between square root mutational target and p_{path} for recessive disease genes previously thought to be affected by balancing selection using FIN allele frequencies. **c)** Observed relationship between square root mutational target and p_{path} for recessive disease genes previously thought to be affected by balancing selection using AFR allele frequencies. **d)** Observed relationship between square root mutational target and p_{path} for recessive disease genes previously thought to be affected by balancing selection using SAS allele frequencies. 43

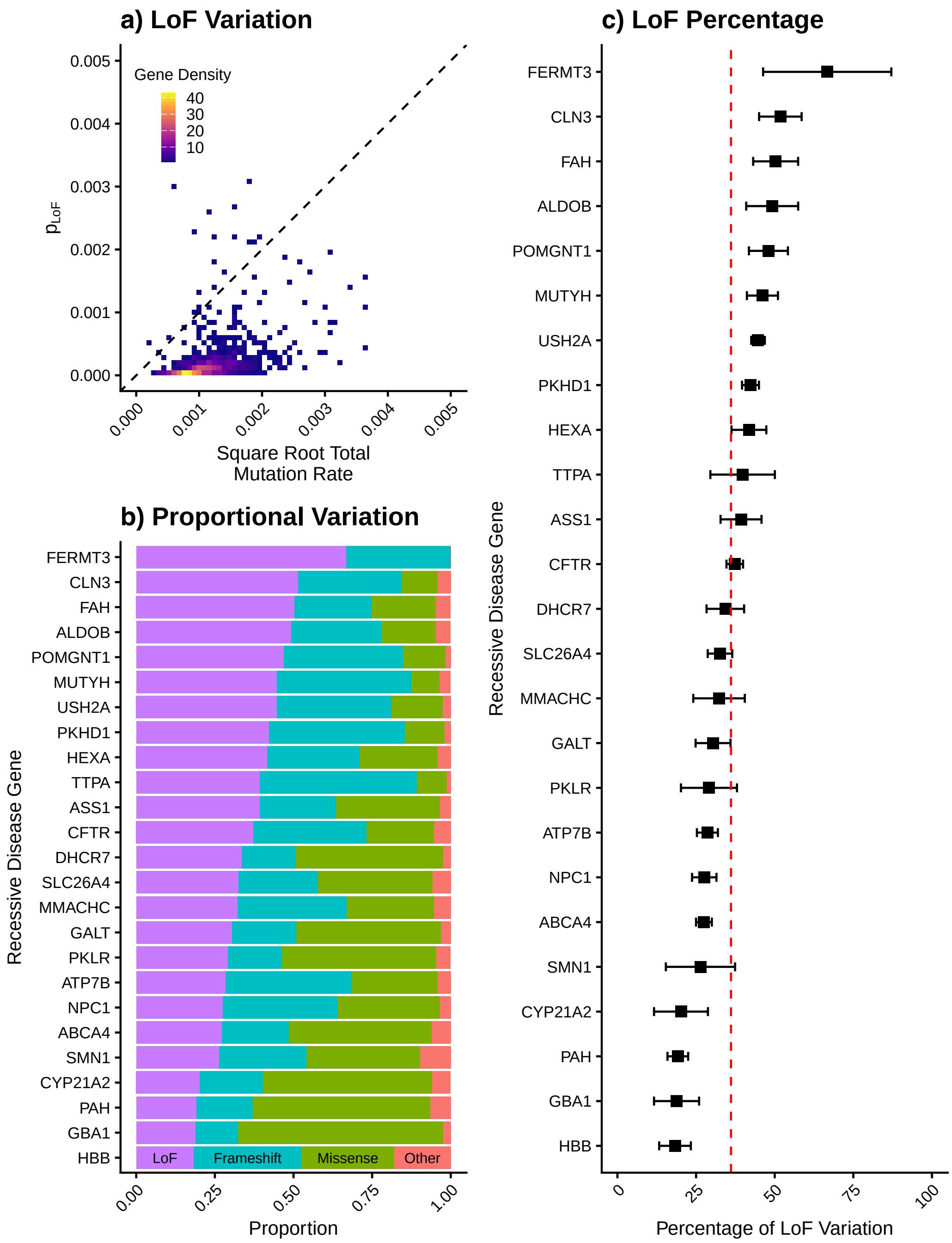

Supplementary Figure 1: LoF and Pathogenic Variation:
a) Density plot for observed relationship between square root mutational target size and p_{LoF} for recessive genes using NFE allele frequencies. **b)** Proportional split of observed pathogenic variation for 25 commonly-studied recessive disease genes based on predicted molecular consequence (LoF, Frameshift, Missense, or Other). **c)** Percentage of observed pathogenic variation for 25 commonly-studied recessive disease genes predicted to be LoFs. Bands represent two standard error confidence intervals surrounding LoF percentage point estimate. Red line represents average percentage of pathogenic variation predicted to be LoFs between all recessive disease genes.

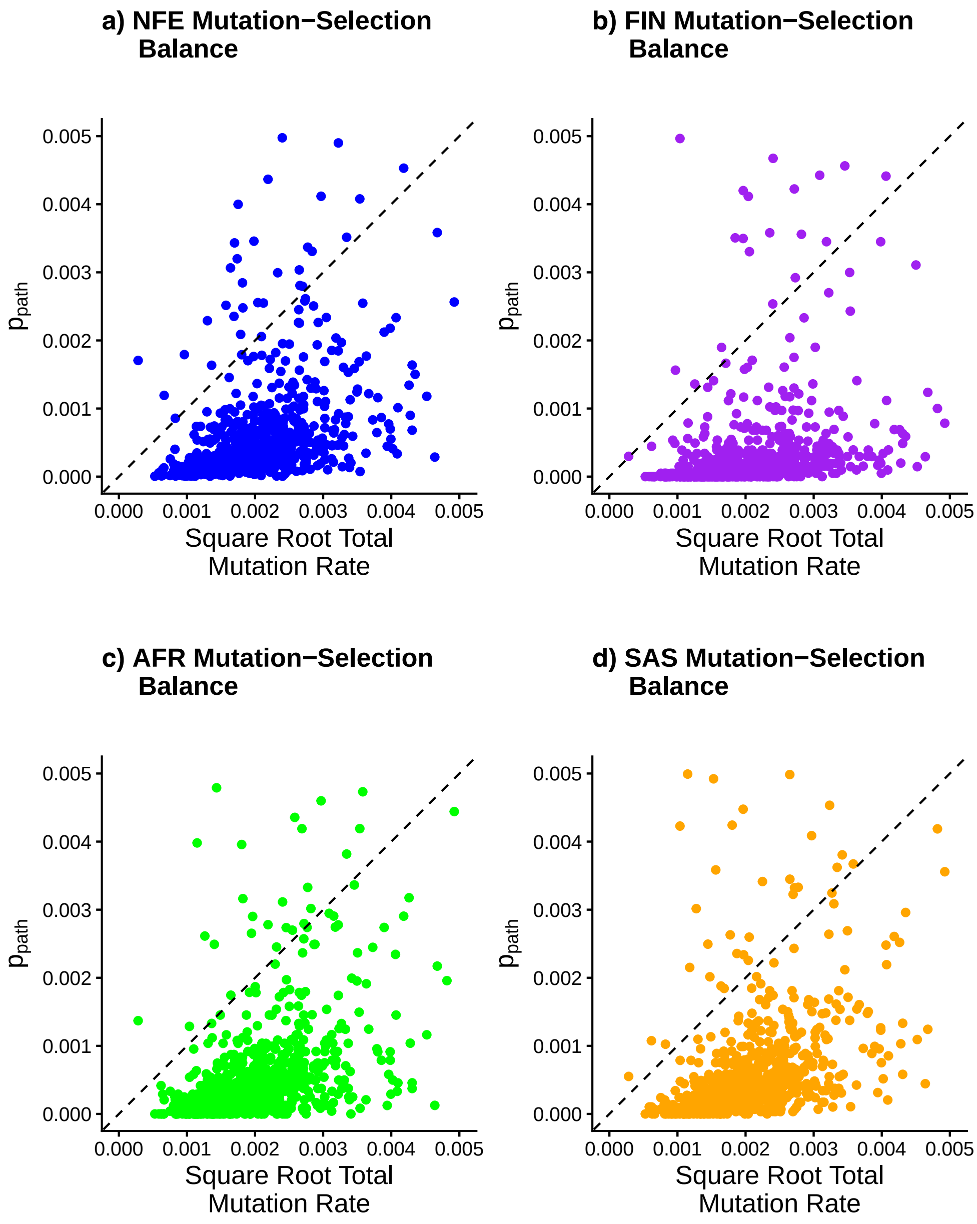

Supplementary Figure 2: Other Population Mutation-Selection Balance:
a) Observed relationship between square root mutational target and p_{path} for recessive genes using NFE allele frequencies. **b)** Observed relationship between square root mutational target and p_{path} for recessive genes using FIN allele frequencies. **c)** Observed relationship between square root mutational target and p_{path} for recessive genes using AFR allele frequencies. **d)** Observed relationship between square root mutational target and p_{path} for recessive genes using SAS allele frequencies.

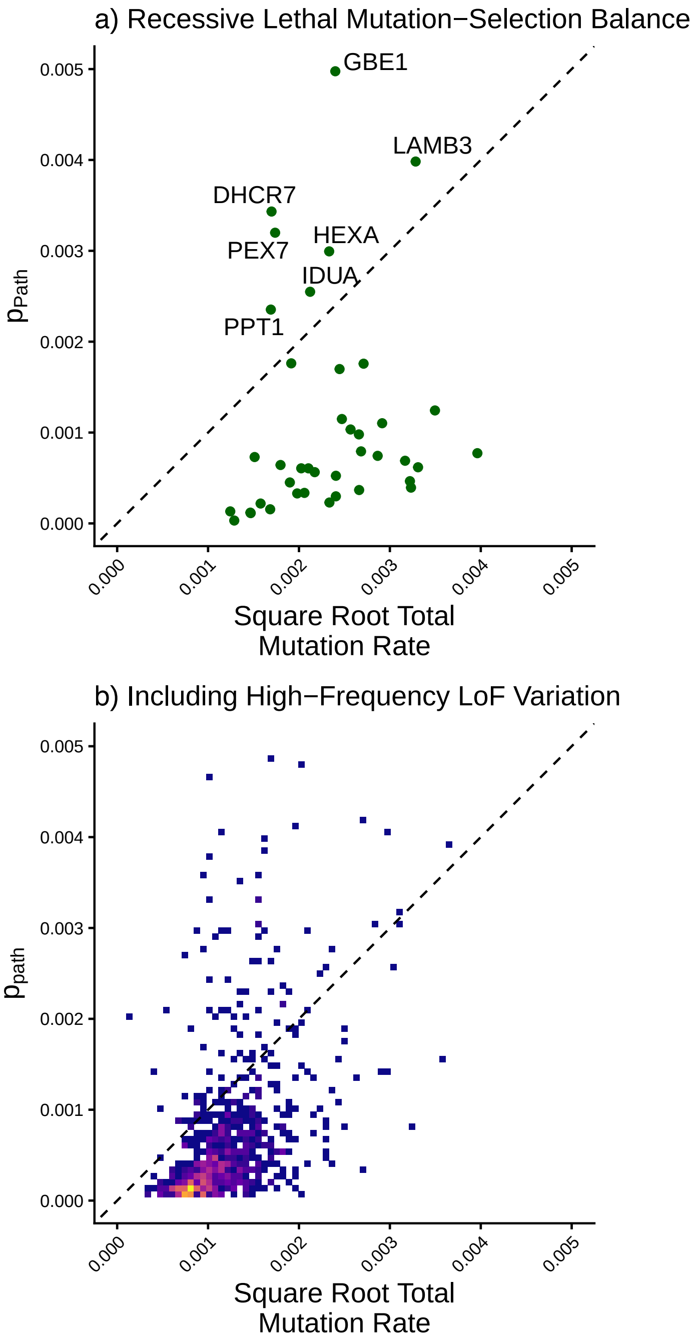

Supplementary Figure 3: Mutation Selection Balance for Recessive Lethal Genes:
a) Observed relationship between square root mutational target size and p_{path} for recessive lethal disease genes. Genes with a predicted p_{path} in the expected range for recessive mutation-selection balance are labeled using non-Finnish European (NFE) allele frequencies. **b)** Observed relationship between square root mutation target size and p_{path} for recessive genes using NFE allele frequencies and not excluding LoF variants with a frequency greater than 0.005 prior to calculating p_{path}.

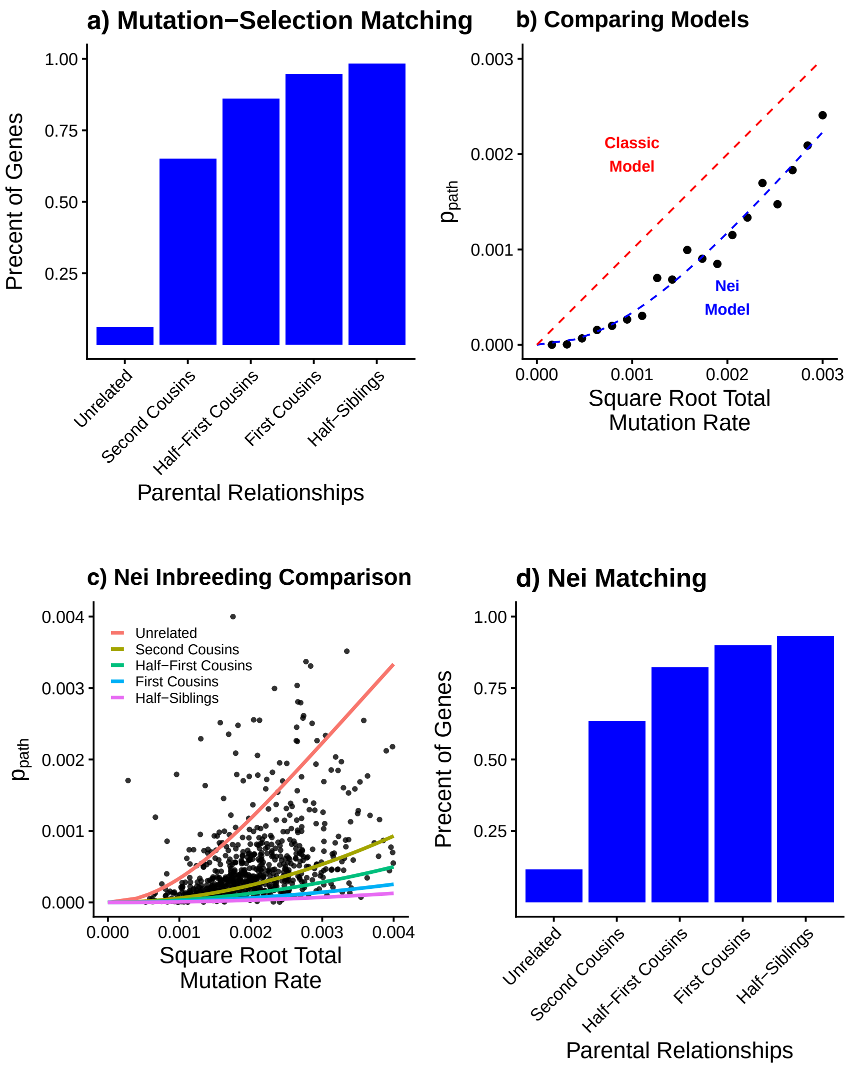

Supplementary Figure 4: Inbreeding Models:
a) Percentage of recessive disease genes that fit the classic recessive mutation-selection balance for different levels of population inbreeding. **b)** Comparison of the classic recessive mutation-selection balance model and adjusted Nei model (Equation 9) for a finite population for populations with no inbreeding. Lines represent predicted p_{path} for a given mutational target size for each model assuming recessive lethality. Dots represent simulated p_{path} data for recessive lethal genes with varying mutational target sizes. **c)** Observed relationship between square root mutational target size and p_{path} for recessive genes using NFE allele frequencies. Lines represent the expected relationship between square root mutational target size and p_{path} under finite population model for different levels of inbreeding in the population. **d)** Percentage of recessive disease genes that fit the finite population model for different levels of population inbreeding.

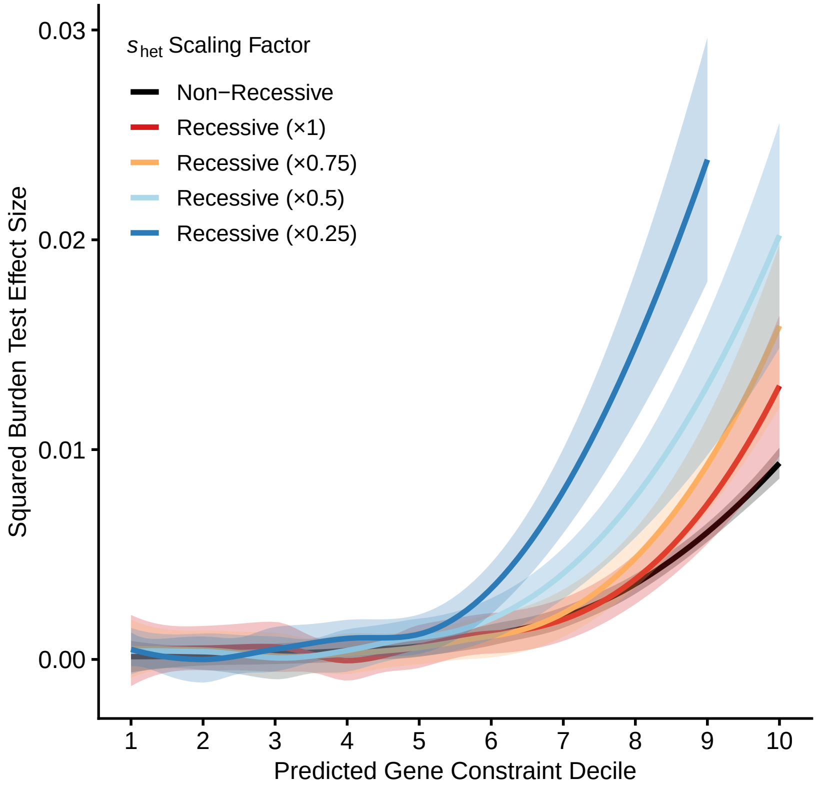

Supplementary Figure 5: Sensitivity of the Enrichment-Constraint Relationship: Relationship between predicted gene constraint decile and average bias-corrected squared LoF burden test effect sizes for 161 quantitative traits. Recessive gene s_{het} values are scaled by factors of $\times 1$, $\times 0.75$, $\times 0.5$, and $\times 0.25$ prior to joint ranking with non-recessive genes into constraint deciles. The non-recessive reference curve (black) is held fixed across all scaling scenarios. Analysis is restricted to the top 75% of genes by mutational target size. Lines show loess curve fits and shaded bands represent 95% confidence intervals.

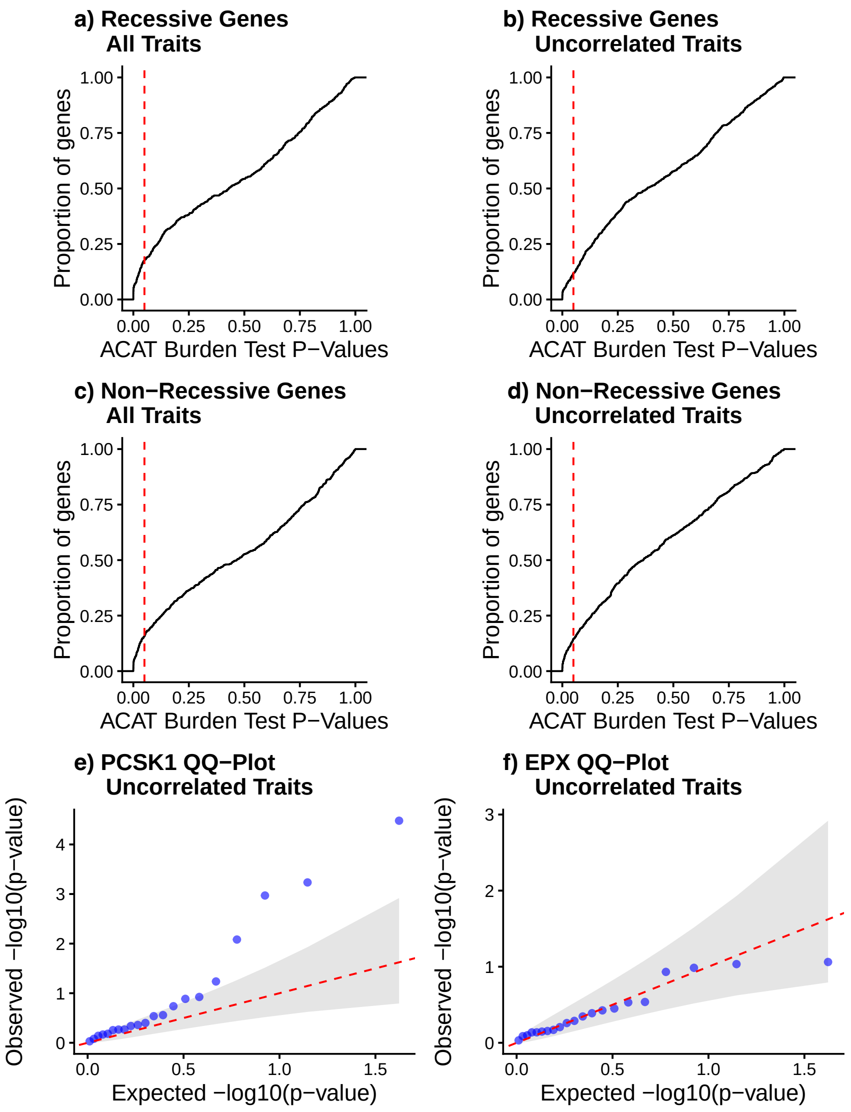

Supplementary Figure 6: Heterozygous Effects:
a) Empirical cumulative distribution function of ACAT omnibus test of p-values from LoF burden tests for 161 UK BioBank (UKB) quantitative traits across 796 recessive disease genes. The vertical line represents p-values that surpass 0.05. **b)** Empirical cumulative distribution function of ACAT omnibus test of p-values from LoF burden tests for 21 uncorrelated UKB quantitative traits across 796 recessive disease genes. The vertical line represents p-values that surpass 0.05. **c)** Empirical cumulative distribution function of ACAT omnibus test of p-values from LoF burden tests for 161 UK BioBank (UKB) quantitative traits across a random sample of 796 non-recessive disease genes. The vertical line represents p-values that surpass 0.05. **d)** Empirical cumulative distribution function of ACAT omnibus test of p-values from LoF burden tests for 21 uncorrelated UKB quantitative traits across a random sample of 796 non-recessive disease genes. The vertical line represents p-values that surpass 0.05. **e)** Quantile-quantile plot of 21 LoF burden test p-values from uncorrelated traits for the recessive *PCSK1* gene. **f)** Quantile-quantile plot of 21 LoF burden test p-values from uncorrelated traits for the recessive *EPX* gene.

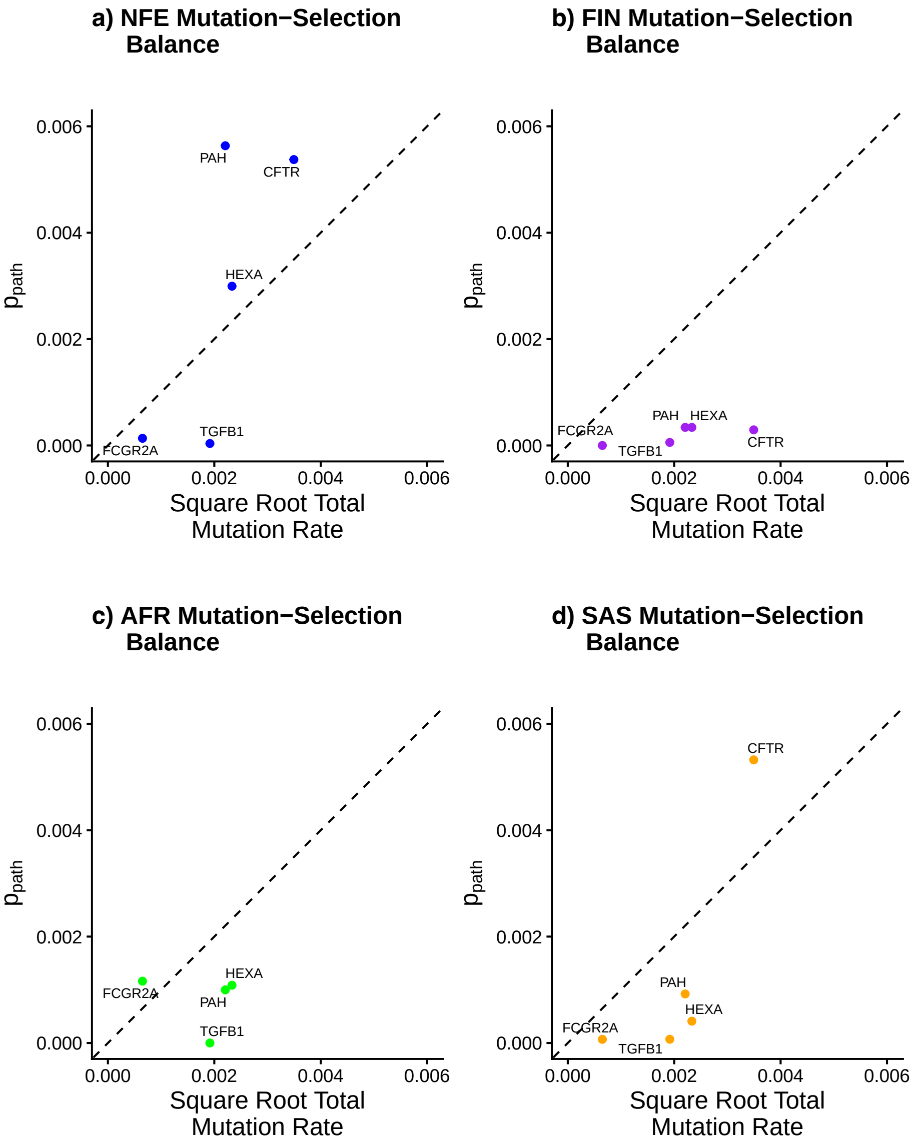

Supplementary Figure 7: Mutation-Selection Balance Under Balancing Selection:
a) Observed relationship between square root mutational target and p_{path} for recessive disease genes previously thought to be affected by balancing selection using NFE allele frequencies. **b)** Observed relationship between square root mutational target and p_{path} for recessive disease genes previously thought to be affected by balancing selection using FIN allele frequencies. **c)** Observed relationship between square root mutational target and p_{path} for recessive disease genes previously thought to be affected by balancing selection using AFR allele frequencies. **d)** Observed relationship between square root mutational target and p_{path} for recessive disease genes previously thought to be affected by balancing selection using SAS allele frequencies.

List of Supplemental Tables

Supplemental Table 1:
List of 796 genes associated with only recessive diseases and no other documented inheritance method listed (in OMIM as of December 2024) (Full Table in Separate File) 46
Supplemental Table 2:
Per-chromosome counts of recessive disease genes (N = 796) compared to all protein-coding genes in the analysis set. Expected counts assume recessive genes are distributed proportionally to protein-coding gene density per chromosome. Enrichment ratio is the observed-to-expected ratio of recessive gene counts. Odds ratios and 95% confidence intervals are from two-sided Fisher exact tests for enrichment on each chromosome versus all others. P-values are Bonferroni-corrected for 22 tests. (Full Table in Separate File) 46
Supplemental Table 3:
Comparison of genomic features between recessive disease genes (N = 796) and non-recessive protein-coding genes. Panel A reports Wilcoxon rank-sum tests comparing feature distributions between gene classes. Panel B reports Spearman rank correlations between genomic features and the estimated proportion of total selection acting through heterozygotes within recessive genes, computed under the assumption of recessive lethality ($s_{hom} = 1$). All p-values are Bonferroni-corrected for the number of features tested within each panel. (Full Table in Separate File) 46
Supplemental Table 4:
List of 25 commonly studied recessive diseases and genes associated with those diseases. For each gene, the number of pathogenic/likely pathogenic variants (identified through ClinVar) is listed as well as the percentage of this variation that is predicted to be loss-of-function (LoF) (nonsense, splice-acceptor disrupting, or splice-donor disrupting), frameshift, missense, or other. For each gene, a conversion factor to account for missing non-LoF pathogenic variation is included and calculated as the inverse and square root inverse of the percentage of LoF variation, respectively. Other variation defined as UTR variants, genic upstream/downstream transcript variants, inframe indels, intron variants, non-coding transcript variants, and synonymous variants . . 47
Supplemental Table 5:
PCSK1 LoF burden test results for 161 non-disease quantitative traits from the UK Biobank. Each row includes effect size estimate, standard error, and p-value for association between PCSK1 LoFs and phenotype (Full Table in Separate File) 48

Supplemental Table 6:
List of recessive genes that appear to follow classic recessive mutation-selection balance based on at least one population-specific pLoF being larger than the gene's square root mutational target size. Square root mutational target size, pLoFs for Non-Finnish European, Finnish, African, and South Asian populations, and populations where the gene matches mutation-selection balance model are included for each gene. 48
Supplemental Table 7:
Gene Ontology enrichment of recessive disease genes in the expected range for recessive mutation-selection balance based on pLoF in Non-Finnish European, Finnish, African, and South Asian populations. Each row identifies the enrichment of purely recessive genes in biological, molecular, and cellular functions and the significance of this enrichment. Background gene list is all recessive disease genes. 52

Supplementary Table 1: Recessive Disease Genes:
List of 796 genes associated with only recessive diseases and no other documented inheritance method listed (in OMIM as of December 2024)
(Full Table in Separate File)

Supplementary Table 2: Chromosomal Distribution:
Per-chromosome counts of recessive disease genes (N = 796) compared to all protein-coding genes in the analysis set. Expected counts assume recessive genes are distributed proportionally to protein-coding gene density per chromosome. Enrichment ratio is the observed-to-expected ratio of recessive gene counts. Odds ratios and 95% confidence intervals are from two-sided Fisher exact tests for enrichment on each chromosome versus all others. P-values are Bonferroni-corrected for 22 tests.
(Full Table in Separate File)

Supplementary Table 3: Genomic Feature Comparisons:
Comparison of genomic features between recessive disease genes (N = 796) and non-recessive protein-coding genes. Panel A reports Wilcoxon rank-sum tests comparing feature distributions between gene classes. Panel B reports Spearman rank correlations between genomic features and the estimated proportion of total selection acting through heterozygotes within recessive genes, computed under the assumption of recessive lethality ($s_{hom} = 1$). All p-values are Bonferroni-corrected for the number of features tested within each panel.
(Full Table in Separate File)

Supplementary Table 4: Classification of Pathogenic/Likely Pathogenic Variation for Studied Recessive Disease Genes: List of 25 commonly studied recessive diseases and genes associated with those diseases. For each gene, the number of pathogenic/likely pathogenic variants (identified through ClinVar) is listed as well as the percentage of this variation that is predicted to be loss-of-function (LoF) (nonsense, splice-acceptor disrupting, or splice-donor discrupting), frameshift, missense, or other. For each gene, a conversion factor to account for missing non-LoF pathogenic variation is included and calculated as the inverse and square root inverse of the percentage of LoF variation, respectively. Other variation defined as UTR variants, genic upstream/downstream transcript variants, inframe indels, intron variants, non-coding transcript variants, and synonymous variants

Genes	Associated_Disease	n_variants	pct_LoF	pct_Frameshift	pct_Missense	pct_Other	pLoF_conversion	sqrtMT_conversion
ABCA4	Stargardt disease	1380	27.5	21.4	45.70	6.14	3.63	1.91
ALDOB	Hereditary fructose intolerance	128	49.2	28.9	17.20	4.69	2.03	1.43
ASS1	Citrullinemia type I	206	39.3	24.3	33.00	3.40	2.54	1.59
ATP7B	Wilson disease	784	28.6	40.2	27.40	4.21	3.50	1.87
CFTR	Cystic fibrosis	1250	37.3	36.1	21.50	5.36	2.68	1.64
CLN3	Juvenile neuronal ceroid lipofuscinosis	191	51.8	33.0	11.50	4.19	1.93	1.39
CYP21A2	Congenital adrenal hyperplasia	119	20.2	20.2	53.80	5.88	4.96	2.23
DHCR7	Smith-Lemli-Opitz syndrome	245	34.3	17.6	47.80	2.45	2.92	1.71
FAH	Tyrosinemia type I	171	50.3	24.6	20.50	4.68	1.99	1.41
FERMT3	Leukocyte adhesion deficiency type III	21	66.7	33.3	0.00	0.00	1.50	1.22
GALT	Classic galactosemia	283	30.4	20.5	45.90	3.18	3.29	1.81
GBA1	Gaucher disease	170	18.8	13.5	65.30	2.35	5.31	2.30
HBB	Sickle cell disease	344	18.3	34.3	29.40	18.00	5.46	2.34
HEXA	Tay-sachs disease	285	41.8	29.5	24.90	4.21	2.39	1.55
MMACHC	Cobalamin C deficiency	130	32.3	34.6	27.70	5.38	3.10	1.76
MUTYH	MUTYH-associated polyposis	358	46.1	44.1	9.22	3.63	2.17	1.47
NPC1	Niemann-Pick disease, type C1	580	27.6	36.4	32.60	3.45	3.63	1.90
PAH	Phenylketonuria (PKU)	808	19.2	17.8	56.40	6.56	5.21	2.28
PKHD1	Autosomal recessive polycystic kidney disease	1200	42.3	43.1	12.70	1.92	2.36	1.54
PKLR	Pyruvate kinase deficiency	110	29.1	17.3	49.10	4.55	3.44	1.85
POMGNT1	Congenital muscular dystrophy	227	48.0	38.8	13.70	1.76	2.08	1.44
SLC26A4	Pendred syndrome, DFNB4 deafness	574	32.6	25.3	36.40	5.75	3.07	1.75
SMN1	Spinal muscular atrophy	72	26.4	27.8	36.10	9.72	3.79	1.95
TTPA	Ataxia with vitamin E deficiency	83	39.8	50.6	9.64	1.20	2.52	1.59
USH2A	Usher syndrome type II	1860	44.7	36.4	16.30	2.59	2.23	1.49

Supplementary Table 5: PCSK1 LoF Burden Tests:
PCSK1 LoF burden test results for 161 non-disease quantitative traits from the UK Biobank. Each row includes effect size estimate, standard error, and p-value for association between PCSK1 LoFs and phenotype
(Full Table in Separate File)

Supplementary Table 6: Potentially Purely Recessive Selection Genes Across Populations: List of recessive genes that appear to follow classic recessive mutation-selection balance based on at least one population-specific pLoF being larger than the gene's square root mutational target size. Square root mutational target size, pLoFs for Non-Finnish European, Finnish, African, and South Asian populations, and populations where the gene matches mutation-selection balance model are included for each gene.

Gene	Square Root Mutational Target Size	Non-Finnish pPath	Finnish pPath	African pPath	South Asian pPath	Potentially Recessive Populations
ABCC2	2.04e-03	4.08e-03	2.43e-03	4.19e-03	7.17e-03	NFE, FIN, AFR, SAS
ABCG5	1.14e-03	1.18e-03	1.17e-03	5.40e-04	2.34e-03	NFE, FIN, SAS
ABCG8	1.26e-03	4.37e-03	8.60e-03	2.78e-03	1.37e-03	NFE, FIN, AFR, SAS
ACADSB	1.05e-03	2.48e-03	1.98e-04	3.16e-03	7.23e-04	NFE, AFR
ADAMTSL4	1.82e-03	6.41e-04	2.45e-04	2.91e-03	7.55e-04	AFR
AGL	2.24e-03	2.12e-03	7.77e-04	2.74e-03	9.92e-04	AFR
AGPAT2	8.26e-04	1.06e-04	0.00e+00	4.79e-03	1.02e-04	AFR
AHI1	1.86e-03	1.85e-03	2.70e-03	2.78e-03	1.69e-03	FIN, AFR
ALG12	1.03e-03	5.61e-04	1.22e-03	3.73e-04	3.41e-04	FIN
ALMS1	1.55e-03	2.79e-03	9.76e-04	2.37e-03	3.23e-03	NFE, AFR, SAS
ALOXE3	1.47e-03	1.22e-03	1.26e-03	6.65e-04	1.54e-03	SAS
ANO6	1.80e-03	1.85e-03	4.86e-04	1.16e-03	1.47e-03	NFE
AP5Z1	1.52e-03	2.26e-03	1.54e-04	1.58e-03	1.35e-03	NFE, AFR
APTX	1.09e-03	1.70e-03	4.86e-05	9.17e-04	3.07e-04	NFE
ARSA	1.14e-03	3.46e-03	1.58e-03	7.06e-04	7.28e-04	NFE, FIN
ASPM	2.97e-03	3.43e-03	2.68e-03	3.04e-03	2.67e-03	NFE, AFR
ATP7B	1.53e-03	1.56e-03	2.04e-03	8.71e-04	3.45e-03	NFE, FIN, SAS
ATPAF2	7.89e-04	1.24e-04	0.00e+00	1.12e-03	6.86e-05	AFR
AUH	1.06e-03	3.81e-04	3.51e-03	2.08e-04	4.14e-04	FIN
BBS2	1.65e-03	2.51e-03	2.33e-03	7.05e-04	6.83e-04	NFE, FIN
BLM	1.52e-03	2.45e-03	6.33e-04	1.33e-03	1.41e-03	NFE
CARD9	1.13e-03	1.98e-04	3.50e-03	2.90e-03	4.48e-03	FIN, AFR, SAS
CATSPER1	9.47e-04	3.32e-04	4.95e-05	1.74e-03	4.11e-04	AFR
CC2D2A	2.45e-03	1.34e-03	6.89e-04	3.18e-03	2.52e-03	AFR, SAS
CD36	1.01e-03	4.00e-03	9.72e-05	7.60e-03	1.11e-02	NFE, AFR, SAS
CD3D	4.74e-04	4.01e-04	0.00e+00	2.49e-04	1.02e-03	SAS
CD3G	6.65e-04	2.84e-04	7.87e-04	2.08e-04	1.02e-04	FIN
CD8A	6.90e-04	1.05e-04	0.00e+00	4.16e-05	7.87e-04	SAS
CEP152	2.06e-03	2.55e-03	3.92e-04	4.73e-03	3.67e-03	NFE, AFR, SAS
CEP290	2.77e-03	5.09e-03	1.00e-03	1.96e-03	4.19e-03	NFE, SAS
CERKL	1.17e-03	2.56e-03	4.12e-03	9.56e-04	2.26e-03	NFE, FIN, SAS
CHRNG	1.35e-03	5.97e-04	1.31e-03	4.56e-04	1.71e-03	SAS
CNGA1	1.37e-03	1.54e-03	1.46e-04	6.65e-04	8.61e-04	NFE
CNGB1	1.68e-03	1.94e-03	2.92e-04	5.89e-03	1.61e-03	NFE, AFR
CNGB3	1.47e-03	8.80e-04	3.89e-04	2.70e-03	6.51e-04	AFR
COG5	1.61e-03	6.35e-04	1.46e-04	5.41e-04	7.35e-03	SAS
COX15	1.14e-03	1.76e-03	1.46e-04	8.72e-04	5.81e-04	NFE
COX4I2	7.22e-04	2.85e-04	1.36e-03	3.32e-04	1.72e-04	FIN
CSTB	3.83e-04	1.19e-03	0.00e+00	2.07e-04	0.00e+00	NFE
CUBN	2.59e-03	5.70e-03	3.11e-03	1.22e-02	6.83e-03	NFE, FIN, AFR, SAS
CUL7	2.15e-03	8.33e-04	1.54e-04	2.45e-03	9.63e-04	AFR
CYP27A1	1.29e-03	1.31e-03	1.46e-04	1.45e-03	3.41e-03	NFE, AFR, SAS

Continued on next page

Gene	Square Root Mutational Target Size	Non-Finnish pPath	Finnish pPath	African pPath	South Asian pPath	Potentially Recessive Populations
CYP2C19	8.07e-04	7.43e-04	6.32e-04	2.49e-03	1.12e-02	AFR, SAS
CYP2R1	1.17e-03	4.85e-04	1.47e-04	1.30e-03	8.04e-04	AFR
CYP4F22	1.18e-03	2.03e-04	3.30e-03	4.57e-04	1.71e-04	FIN
CYP4V2	1.13e-03	6.80e-04	4.20e-03	4.98e-04	3.42e-04	FIN
D2HGDH	1.01e-03	1.43e-04	4.92e-04	1.04e-03	3.76e-04	AFR
DARS2	1.41e-03	9.90e-04	5.39e-03	1.37e-03	5.46e-04	FIN
DBT	1.12e-03	1.19e-04	4.86e-05	2.65e-03	1.37e-04	AFR
DCLRE1C	1.15e-03	6.54e-04	1.46e-04	1.87e-03	3.76e-04	AFR
DDB2	1.17e-03	2.79e-04	1.60e-03	4.15e-05	3.41e-05	FIN
DHCR7	9.78e-04	3.43e-03	6.52e-03	5.44e-03	1.20e-03	NFE, FIN, AFR, SAS
DMGDH	1.56e-03	6.85e-03	4.22e-03	2.57e-03	8.81e-03	NFE, FIN, AFR, SAS
DNAAF1	1.63e-04	1.71e-03	2.97e-04	1.37e-03	5.51e-04	NFE, FIN, AFR, SAS
DNAH11	3.61e-03	4.93e-03	3.07e-03	3.42e-03	5.71e-03	NFE, SAS
DNAH5	3.65e-03	3.34e-03	7.82e-03	3.20e-03	3.18e-03	FIN
DNAI1	1.33e-03	1.82e-03	6.80e-04	9.54e-04	2.73e-04	NFE
DNAI2	1.39e-03	7.31e-04	1.96e-04	1.78e-03	2.22e-03	AFR, SAS
DPYD	1.65e-03	1.63e-02	2.44e-04	2.49e-03	1.33e-02	NFE, AFR, SAS
DPYS	1.21e-03	1.78e-03	9.71e-05	4.98e-04	8.88e-04	NFE
DSG4	1.55e-03	7.07e-04	1.95e-04	4.19e-03	6.85e-04	AFR
DUOX2	1.93e-03	3.52e-03	3.89e-04	3.82e-03	3.62e-03	NFE, AFR, SAS
DUOXA2	7.54e-04	5.46e-04	9.83e-05	1.04e-03	7.53e-04	AFR
DYSF	2.50e-03	1.50e-03	5.90e-04	6.15e-03	2.96e-03	AFR, SAS
EIF2B1	8.99e-04	8.33e-04	4.86e-05	7.05e-04	3.58e-03	SAS
ENO3	1.08e-03	5.43e-04	0.00e+00	1.45e-03	2.36e-03	AFR, SAS
EPX	1.58e-03	1.22e-02	7.10e-03	1.04e-02	2.53e-02	NFE, FIN, AFR, SAS
ERCC3	1.66e-03	1.39e-03	1.46e-04	2.49e-03	8.88e-04	AFR
ERCC4	1.44e-03	4.39e-04	1.05e-04	1.58e-03	7.17e-04	AFR
EYS	1.71e-03	4.12e-03	1.12e-03	4.60e-03	4.09e-03	NFE, AFR, SAS
FAM161A	7.49e-04	2.29e-03	1.94e-04	4.98e-04	1.10e-03	NFE, SAS
FANCA	2.03e-03	1.69e-03	3.00e-03	1.49e-03	1.37e-03	FIN
FANCC	1.27e-03	1.59e-03	0.00e+00	1.45e-03	1.06e-03	NFE, AFR
FANCD2	1.57e-03	2.58e-03	2.92e-03	1.34e-03	1.13e-03	NFE, FIN
FANCM	1.97e-03	6.90e-03	1.07e-02	1.99e-03	3.81e-03	NFE, FIN, AFR, SAS
FASTKD2	9.84e-04	5.11e-04	1.66e-03	2.50e-04	5.83e-04	FIN
FKRP	8.31e-04	1.12e-04	8.79e-04	4.15e-05	2.19e-04	FIN
FMO3	9.44e-04	3.07e-03	4.86e-05	5.81e-04	1.88e-03	NFE, SAS
FOLR1	8.81e-04	6.98e-03	1.41e-03	1.04e-03	6.83e-05	NFE, FIN, AFR
FOXRED1	1.27e-03	7.53e-04	1.00e-04	4.16e-04	1.68e-03	SAS
FTCD	1.22e-03	7.53e-04	2.71e-04	1.41e-02	8.95e-04	AFR
FTO	1.34e-03	2.69e-04	1.94e-04	3.33e-04	1.37e-03	SAS
GAA	1.54e-03	7.93e-04	8.31e-04	7.06e-03	1.24e-03	AFR
GALC	1.48e-03	1.03e-03	1.61e-03	5.42e-04	7.24e-04	FIN
GALNT3	1.29e-03	4.44e-04	3.89e-04	5.52e-03	3.43e-04	AFR
GAMT	6.91e-04	7.38e-04	0.00e+00	4.15e-04	1.02e-04	NFE
GBE1	1.38e-03	4.98e-03	2.54e-03	8.70e-04	6.03e-04	NFE, FIN
GNPTG	1.03e-03	1.05e-03	1.46e-04	6.64e-04	4.13e-04	NFE
GP1BB	3.55e-04	1.33e-05	0.00e+00	0.00e+00	1.08e-03	SAS
GPX1	3.57e-04	5.03e-05	4.45e-04	4.16e-04	1.03e-04	FIN, AFR
GRHPR	9.48e-04	6.54e-04	1.90e-03	8.72e-04	5.13e-04	FIN
GRM6	1.36e-03	1.37e-03	3.58e-03	6.22e-04	1.81e-03	NFE, FIN, SAS
GYS2	1.58e-03	2.61e-03	2.92e-04	9.16e-04	8.16e-03	NFE, SAS
HAX1	9.09e-04	3.07e-04	3.89e-04	1.16e-03	2.73e-04	AFR
HEXA	1.34e-03	2.99e-03	3.40e-04	1.09e-03	4.10e-04	NFE
HGSNAT	1.44e-03	6.50e-04	5.02e-05	1.83e-03	4.58e-04	AFR
HMGCS2	1.32e-03	7.20e-04	0.00e+00	2.20e-03	1.60e-03	AFR, SAS
HOGA1	5.98e-04	2.77e-04	0.00e+00	5.39e-04	7.87e-04	SAS
IDUA	1.22e-03	2.55e-03	6.66e-03	4.15e-04	2.08e-04	NFE, FIN
IGHMBP2	1.69e-03	2.26e-03	9.30e-04	7.47e-04	1.68e-03	NFE
IL12RB1	1.53e-03	2.25e-03	3.96e-04	1.29e-03	1.28e-03	NFE
IRAK4	1.05e-03	2.85e-03	4.88e-04	8.30e-04	3.43e-04	NFE
ITGA7	1.88e-03	1.97e-03	4.42e-04	1.33e-03	3.25e-03	NFE, SAS
KCNV2	8.58e-04	9.31e-04	2.47e-04	1.45e-03	1.13e-03	NFE, AFR, SAS

Continued on next page

Gene	Square Root Mutational Target Size	Non-Finnish pPath	Finnish pPath	African pPath	South Asian pPath	Potentially Recessive Populations
KHK	9.71e-04	7.27e-04	4.87e-05	4.14e-05	1.84e-03	SAS
KLK4	6.63e-04	2.24e-04	0.00e+00	7.00e-03	2.08e-04	AFR
KLKB1	1.24e-03	8.84e-04	9.76e-05	6.64e-04	2.02e-03	SAS
KRT8	9.33e-04	1.46e-03	3.40e-04	4.57e-04	3.42e-04	NFE
LAMA2	3.09e-03	2.59e-03	9.78e-04	2.84e-03	3.21e-03	SAS
LMBRD1	1.33e-03	4.02e-04	4.86e-05	2.45e-03	7.63e-04	AFR
LOXHD1	2.34e-03	5.87e-03	4.41e-03	2.34e-03	2.48e-03	NFE, FIN, AFR, SAS
LPIN1	1.58e-03	6.40e-04	1.46e-04	1.80e-03	9.77e-04	AFR
LTC4S	5.97e-04	9.41e-03	4.97e-03	1.29e-03	4.23e-03	NFE, FIN, AFR, SAS
MAN2B1	1.62e-03	1.30e-03	3.56e-03	3.02e-03	1.20e-03	FIN, AFR
MARVELD2	1.28e-03	1.72e-03	9.77e-05	7.88e-04	1.91e-03	NFE, SAS
MASP2	1.21e-03	8.00e-04	2.45e-04	1.04e-03	1.24e-03	SAS
MCEE	5.54e-04	1.79e-03	4.87e-04	2.49e-04	0.00e+00	NFE
MMAA	1.21e-03	2.06e-03	3.40e-04	4.99e-04	1.48e-03	NFE, SAS
MMAB	7.71e-04	5.01e-04	5.27e-05	1.66e-04	9.56e-04	SAS
MOCS2	5.59e-04	9.76e-05	1.56e-03	8.29e-05	6.83e-05	FIN
MPV17	7.27e-04	9.49e-05	4.86e-05	2.61e-03	2.05e-04	AFR
MPZL2	8.33e-04	7.06e-04	4.86e-05	7.47e-04	2.49e-03	SAS
MS4A1	6.33e-04	1.14e-04	0.00e+00	9.53e-04	4.44e-04	AFR
MUTYH	1.23e-03	8.25e-04	1.46e-04	7.88e-04	6.86e-03	SAS
MYO15A	1.60e-03	3.37e-03	9.69e-04	3.33e-03	3.33e-03	NFE, AFR, SAS
NBN	1.24e-03	6.01e-04	6.80e-04	9.58e-04	1.33e-03	SAS
NDRG1	1.21e-03	3.40e-04	1.71e-03	8.30e-05	3.13e-04	FIN
NDUFAF2	6.55e-04	7.37e-04	0.00e+00	6.35e-04	2.07e-04	NFE
NDUFAF3	6.79e-04	5.53e-05	0.00e+00	4.14e-05	2.15e-03	SAS
NDUFAF5	9.39e-04	9.96e-04	9.76e-05	2.08e-04	7.56e-04	NFE
NDUFAF6	1.02e-03	4.34e-04	3.90e-04	4.60e-04	2.63e-03	SAS
NEB	3.12e-03	6.16e-03	3.88e-03	3.27e-03	3.51e-03	NFE, FIN, AFR, SAS
NME8	1.20e-03	1.05e-03	2.43e-04	1.34e-02	1.85e-03	AFR, SAS
NPC2	6.62e-04	5.38e-04	8.50e-03	3.98e-03	4.99e-03	FIN, AFR, SAS
NPHP3	1.41e-03	1.70e-03	1.02e-03	7.49e-04	7.54e-04	NFE
NPHP4	2.01e-03	1.24e-03	2.96e-04	1.95e-03	2.69e-03	SAS
NPHS1	1.84e-03	2.04e-03	3.45e-03	7.89e-04	1.09e-03	NFE, FIN
NT5E	1.09e-03	6.45e-04	0.00e+00	1.20e-03	4.45e-04	AFR
OBSL1	1.83e-03	9.70e-03	1.39e-02	2.74e-03	1.48e-03	NFE, FIN, AFR
OCA2	1.42e-03	3.75e-04	0.00e+00	2.74e-03	2.39e-04	AFR
OTOA	1.04e-03	1.79e-03	2.93e-04	3.96e-03	4.24e-03	NFE, AFR, SAS
OTOF	2.41e-03	4.53e-03	6.91e-04	2.90e-03	2.61e-03	NFE, AFR, SAS
P3H1	1.45e-03	5.57e-04	2.91e-04	7.95e-03	7.85e-04	AFR
PAH	1.27e-03	5.63e-03	3.40e-04	9.98e-04	9.22e-04	NFE
PCBD1	4.77e-04	8.56e-04	0.00e+00	2.07e-04	3.41e-05	NFE
PCK2	1.49e-03	2.50e-02	1.18e-03	4.36e-03	6.01e-03	NFE, AFR, SAS
PCNT	2.84e-03	2.56e-03	7.84e-04	4.44e-03	3.56e-03	AFR, SAS
PDE6A	1.54e-03	6.86e-04	1.95e-04	1.74e-03	1.81e-03	AFR, SAS
PEX7	1.00e-03	3.20e-03	4.37e-04	1.08e-03	2.05e-04	NFE, AFR
PHKB	1.86e-03	6.69e-03	1.94e-04	1.25e-03	4.53e-03	NFE, SAS
PHYH	8.32e-04	2.93e-04	1.31e-03	4.17e-04	1.37e-04	FIN
PKHD1	3.05e-03	2.18e-03	4.38e-04	2.50e-03	4.45e-03	SAS
PLOD2	1.57e-03	5.88e-04	6.81e-03	3.78e-04	6.56e-04	FIN
PMM2	8.98e-04	9.82e-04	2.45e-04	2.50e-04	7.53e-04	NFE
PNKP	1.20e-03	8.14e-04	9.72e-05	8.33e-04	1.21e-03	SAS
PPT1	9.73e-04	2.35e-03	9.72e-05	6.23e-04	2.05e-04	NFE
PRSS12	1.53e-03	3.04e-03	1.17e-03	1.78e-03	4.98e-03	NFE, AFR, SAS
PYGL	1.59e-03	1.42e-03	3.07e-04	2.74e-03	1.16e-03	AFR
RAB23	9.06e-04	2.52e-03	4.37e-04	4.98e-04	1.03e-04	NFE
RPGRIP1	1.36e-03	1.15e-03	1.02e-03	1.72e-03	1.21e-03	AFR
RPGRIP1L	2.02e-03	1.28e-03	5.83e-04	2.37e-03	1.71e-03	AFR
RSPH4A	1.17e-03	9.94e-04	3.89e-04	2.91e-04	1.33e-03	SAS
SGSH	1.01e-03	3.16e-04	1.12e-03	3.32e-04	1.03e-04	FIN
SLC10A2	7.84e-04	1.63e-03	0.00e+00	1.33e-03	2.06e-04	NFE, AFR
SLC12A3	1.63e-03	3.31e-03	1.48e-04	1.46e-03	8.57e-04	NFE
SLC22A12	1.18e-03	1.90e-04	1.04e-04	2.90e-04	2.60e-03	SAS

Continued on next page

Gene	Square Root Mutational Target Size	Non-Finnish pPath	Finnish pPath	African pPath	South Asian pPath	Potentially Recessive Populations
SLC25A13	1.56e-03	1.18e-03	0.00e+00	6.67e-04	2.43e-03	SAS
SLC25A20	1.10e-03	3.70e-04	4.86e-05	1.79e-03	2.73e-04	AFR
SLC29A3	8.50e-04	3.37e-04	4.86e-05	1.66e-04	2.01e-03	SAS
SLC34A3	1.03e-03	2.09e-03	5.46e-04	5.41e-04	1.06e-03	NFE, SAS
SLC6A19	1.38e-03	1.95e-03	4.67e-03	3.11e-03	1.74e-03	NFE, FIN, AFR, SAS
SLC7A7	1.08e-03	2.90e-04	1.33e-02	3.73e-04	8.53e-04	FIN
SMPD1	1.09e-03	4.51e-04	9.82e-05	1.24e-04	1.43e-03	SAS
SP110	8.81e-04	9.30e-04	1.94e-04	7.47e-04	4.92e-03	NFE, SAS
SPATA7	1.09e-03	7.09e-04	3.88e-04	1.08e-03	1.37e-03	SAS
SPINK5	1.74e-03	1.69e-03	1.90e-03	1.04e-03	1.20e-03	FIN
SRD5A3	7.35e-04	1.35e-04	4.99e-05	1.66e-04	3.02e-03	SAS
STXBP2	1.17e-03	1.37e-03	9.74e-05	7.58e-04	7.56e-04	NFE
SUMF1	1.05e-03	1.50e-04	0.00e+00	1.12e-03	3.10e-04	AFR
TBX19	9.90e-04	4.40e-04	5.30e-03	4.56e-04	6.83e-05	FIN
TCIRG1	1.56e-03	1.76e-03	1.30e-03	3.75e-04	9.34e-04	NFE
TGM1	1.53e-03	2.81e-03	5.88e-04	9.95e-04	5.81e-04	NFE
TMC6	1.42e-03	4.25e-04	0.00e+00	1.97e-03	6.72e-04	AFR
TMEM67	1.75e-03	2.34e-03	3.90e-04	1.08e-03	1.24e-03	NFE
TMEM70	7.45e-04	9.52e-04	4.85e-05	2.91e-04	6.83e-05	NFE
TMPRSS15	1.57e-03	8.22e-03	2.43e-04	2.80e-03	3.32e-03	NFE, AFR, SAS
TPP1	1.21e-03	6.82e-03	6.81e-04	7.05e-04	2.39e-04	NFE
TRIOBP	2.69e-03	3.58e-03	1.24e-03	2.17e-03	1.24e-03	NFE
TRIP11	2.29e-03	2.18e-03	3.45e-03	9.13e-04	1.27e-03	FIN
TYMP	9.91e-04	1.22e-03	4.99e-05	4.15e-04	3.76e-04	NFE
TYRP1	1.03e-03	7.51e-04	2.92e-04	5.65e-03	5.80e-04	AFR
UNC13D	1.90e-03	1.60e-03	3.44e-04	4.17e-04	3.09e-03	SAS
UPB1	1.16e-03	8.13e-03	5.93e-03	1.78e-03	1.05e-02	NFE, FIN, AFR, SAS
UROC1	1.33e-03	6.52e-04	1.47e-04	1.54e-03	8.60e-04	AFR
USH1C	1.44e-03	1.95e-03	2.43e-04	9.30e-03	8.89e-04	NFE, AFR
USH2A	3.41e-03	4.27e-03	2.38e-03	4.11e-03	5.46e-03	NFE, AFR, SAS
WDR35	1.99e-03	1.59e-03	4.56e-03	3.36e-03	2.12e-03	FIN, AFR, SAS
WDR72	1.56e-03	9.97e-04	1.75e-03	1.09e-03	1.71e-03	FIN, SAS
WRN	1.78e-03	6.60e-03	4.43e-03	2.95e-03	1.27e-03	NFE, FIN, AFR
XDH	1.86e-03	4.90e-03	4.86e-04	1.74e-03	2.64e-03	NFE, SAS
XPA	9.76e-04	3.01e-04	9.25e-03	6.65e-04	2.73e-04	FIN

Supplementary Table 7: Select Recessive Genes GO Enrichment:
Gene Ontology enrichment of recessive disease genes in the expected range for recessive mutation-selection balance based on pLoF in Non-Finnish European, Finnish, African, and South Asian populations. Each row identifies the enrichment of purely recessive genes in biological, molecular, and cellular functions and the significance of this enrichment. Background gene list is all recessive disease genes.

Population	Ontology	ID	Description	GeneRatio	BgRatio	p-value	p.adjust	qvalue	Count	geneID
nfe	CC	GO:0016324	apical plasma membrane	.16/94	45/796	1.53e-05	1.58e-03	1.45e-03	16	ABCC2/ABCG5/ABCG8/ ASPM/CD36/CUBN/ DUOX2/FOLR1/MARVELD2/ OTOA/SLC10A2/SLC12A3/ SLC34A3/SLC6A19/TCIRG1/ USH2A
nfe	CC	GO:0045177	apical part of cell	.17/94	50/796	1.59e-05	1.58e-03	1.45e-03	17	ABCC2/ABCG5/ABCG8/ ASPM/CD36/CUBN/ DUOX2/FOLR1/MARVELD2/ OTOA/SLC10A2/SLC12A3/ SLC34A3/SLC6A19/TCIRG1/ USH1C/USH2A
nfe	CC	GO:0098862	cluster of actin-based cell projections	.12/94	32/796	1.13e-04	7.44e-03	6.85e-03	12	BBS2/CD36/CUBN/ FOLR1/LOXHD1/MYO15A/ SLC34A3/SLC6A19/TMPRSS15/ TRIOBP/USH1C/USH2A
nfe	CC	GO:0032420	stereocilium	.6/94	10/796	3.30e-04	1.64e-02	1.50e-02	6	BBS2/LOXHD1/MYO15A/ TRIOBP/USH1C/USH2A
nfe	CC	GO:0042995	cell projection	.31/94	154/796	5.76e-04	2.28e-02	2.10e-02	31	ALMS1/BBS2/CD36/ CEP290/CERKL/CNGA1/ CNGB1/CUBN/DNAAF1/ DNAH11/DNAI1/EYS/ FAM161A/FOLR1/GRM6/ IGHMBP2/LOXHD1/MYO15A/ NPHP3/NPHS1/OTOF/ PPT1/PRSS12/SLC10A2/ SLC34A3/SLC6A19/TMEM67/ TRIOBP/USH1C/USH2A/WRN
nfe	CC	GO:0098590	plasma membrane region	.22/94	97/796	8.65e-04	2.85e-02	2.62e-02	22	ABCC2/ABCG5/ABCG8/ ASPM/ATP7B/BBS2/ CD36/CNGA1/CNGB1/ CUBN/DUOX2/FOLR1/ MARVELD2/OTOA/OTOF/ SLC10A2/SLC12A3/SLC34A3/ SLC6A19/TCIRG1/TMEM67/ USH2A

Continued on next page

Population	Ontology	ID	Description	GeneRatio	BgRatio	p-value	p.adjust	qvalue	Count	geneID
nfe	CC	GO:0032421	stereocilium bundle	.6/94	12/796	1.19e-03	3.37e-02	3.10e-02	6	BBS2/LOXHD1/MYO15A/ TRIOBP/USH1C/USH2A
nfe	CC	GO:0098858	actin-based cell projection	.8/94	21/796	1.56e-03	3.77e-02	3.47e-02	8	BBS2/CUBN/LOXHD1/ MYO15A/SLC10A2/TRIOBP/ USH1C/USH2A
nfe	CC	GO:0120025	plasma membrane bounded cell projection	.29/94	149/796	1.71e-03	3.77e-02	3.47e-02	29	ALMS1/BBS2/CD36/ CEP290/CERKL/CNGA1/ CNGB1/CUBN/DNAAF1/ DNAH11/DNAI1/EYS/ FAM161A/FOLR1/GRM6/ IGHMBP2/LOXHD1/MYO15A/ NPHP3/PPT1/PRSS12/ SLC10A2/SLC34A3/SLC6A19/ TMEM67/TRIOBP/USH1C/ USH2A/WRN
nfe	MF	GO:0016462	pyrophosphatase activity	.10/92	26/783	3.35e-04	2.01e-02	1.95e-02	10	ABCG5/ABCG8/APTX/ ATP7B/BLM/FANCM/ IGHMBP2/MMAA/RAB23/ WRN
nfe	MF	GO:0016817	hydrolase activity, acting on acid anhydrides	.10/92	26/783	3.35e-04	2.01e-02	1.95e-02	10	ABCG5/ABCG8/APTX/ ATP7B/BLM/FANCM/ IGHMBP2/MMAA/RAB23/ WRN
nfe	MF	GO:0016818	hydrolase activity, acting on acid anhydrides, in phosphorus-containing anhydrides	.10/92	26/783	3.35e-04	2.01e-02	1.95e-02	10	ABCG5/ABCG8/APTX/ ATP7B/BLM/FANCM/ IGHMBP2/MMAA/RAB23/ WRN
